# Critical review on in silico methods for structural annotation of chemicals detected with LC/HRMS non-targeted screening

**DOI:** 10.1007/s00216-024-05471-x

**Published:** 2024-08-14

**Authors:** Henrik Hupatz, Ida Rahu, Wei-Chieh Wang, Pilleriin Peets, Emma H. Palm, Anneli Kruve

**Affiliations:** 1 Department of Materials and Environmental Chemistry, Stockholm University, Svante Arrhenius Väg 16, 114 18 Stockholm, Sweden; 2 Stockholm University Center for Circular and Sustainable Systems (SUCCeSS), Stockholm University, 106 91 Stockholm, Sweden; 3 Institute of Biodiversity, Faculty of Biological Science, Cluster of Excellence Balance of the Microverse, Friedrich Schiller University Jena, 07743 Jena, Germany; 4 Luxembourg Centre for Systems Biomedicine (LCSB), University of Luxembourg, 6 Avenue du Swing, 4367 Belvaux, Luxembourg; 5 Department of Environmental Science, Stockholm University, Svante Arrhenius Väg 8, 114 18 Stockholm, Sweden

**Keywords:** Untargeted screening, Suspect screening, Non-targeted analysis, Non-targeted screening, Machine learning, Generative modeling

## Abstract

Non-targeted screening with liquid chromatography coupled to high-resolution mass spectrometry (LC/HRMS) is increasingly leveraging in silico﻿ methods, including machine learning, to obtain candidate structures for structural annotation of LC/HRMS features and their further prioritization. Candidate structures are commonly retrieved based on the tandem mass spectral information either from spectral or structural databases; however, the vast majority of the detected LC/HRMS features remain unannotated, constituting what we refer to as a part of the unknown chemical space. Recently, the exploration of this chemical space has become accessible through generative models. Furthermore, the evaluation of the candidate structures benefits from the complementary empirical analytical information such as retention time, collision cross section values, and ionization type. In this critical review, we provide an overview of the current approaches for retrieving and prioritizing candidate structures. These approaches come with their own set of advantages and limitations, as we showcase in the example of structural annotation of ten known and ten unknown LC/HRMS features. We emphasize that these limitations stem from both experimental and computational considerations. Finally, we highlight three key considerations for the future development of in silico methods.

**Graphical Abstract:**

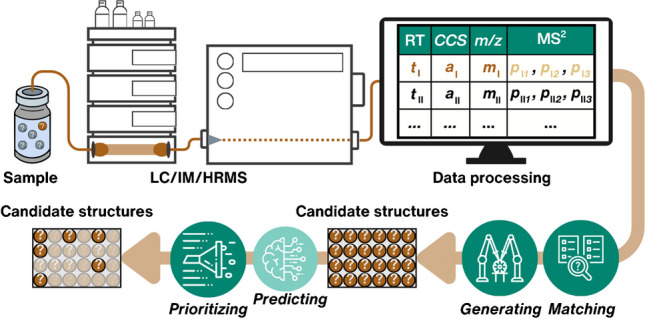

**Supplementary Information:**

The online version contains supplementary material available at 10.1007/s00216-024-05471-x.

## Introduction

**Fig. 1:**
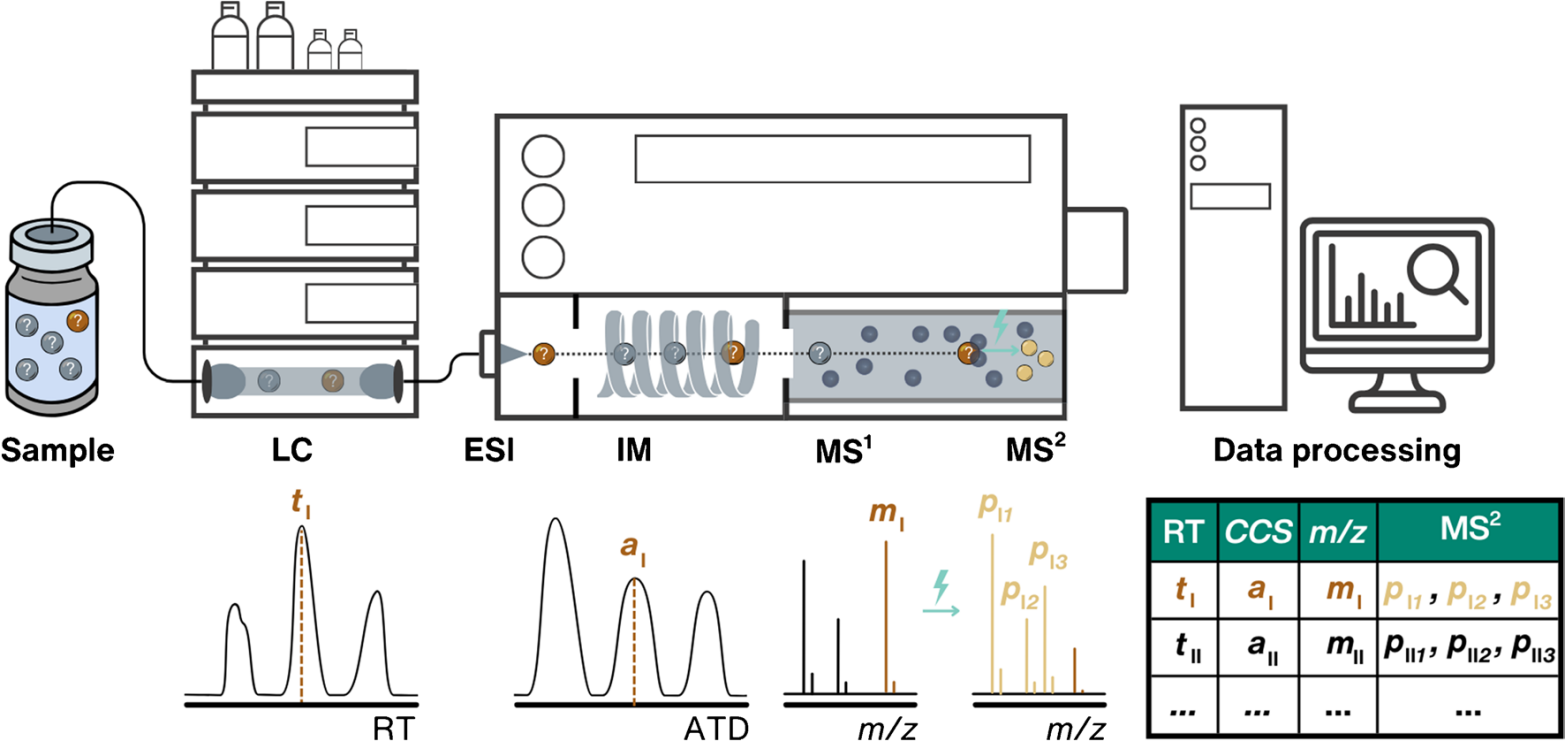
Experimental workflow for analyzing an environmental sample using LC/IM/HRMS experiment with electrospray ionization (ESI). Dark brown indicates experimental analytical features (RT *t*_I_, *CCS a*_I_, and *m/z m*_I_) of an unknown structure, while light brown marks its MS^2^ features (*p*_I*1*_, *p*_I*2*,_ and *p*_I*3*_). *CCS* values are derived from arrival time distributions (ATD). The schematical table with analytical information will appear in subsequent figures to highlight the in silico structural annotation workflow for LC/HRMS features

Non-targeted screening (NTS) is a theoretical concept of detecting and identifying a wide range of chemicals in complex samples with minimal prior information, increasingly applied in environmental monitoring. In practice, the identifiable chemicals are restricted by the ones extracted with the sample preparation method, retained and separated in liquid chromatography (LC) and potentially in ion mobility (IM), and detected by high-resolution mass spectrometry (HRMS) (Fig. 1) [1]. The success depends on the data processing workflows: peak detection, alignment, blank correction, etc. [2]. Finally, empirical analytical information can be used to obtain candidate structures for structural annotation and prioritize them, while identification can be achieved by directly comparing the acquired analytical information with that of analytical standards. Underperformance in any of these steps is detrimental to the outcome of NTS. This review particularly focuses on in silico methods for candidate structure retrieval and prioritization.

NTS commonly detects thousands of LC/HRMS features, i.e., a combination of the accurate mass and retention time (RT), with or without a tandem mass spectrum (MS^2^). Matching the experimental spectral information with a structure poses a significant challenge and becomes overwhelming if manual data curation is required. Workflows for the structural annotation of the chemicals start with obtaining the candidate structures based on the full scan (MS^1^) and MS^2^ either by matching the spectra of the unknown LC/HRMS features with the experimental or in silico spectra in spectral libraries (“Library﻿ MS2 spectra matching” and “In silico MS2 spectra matching” sections), automated interpretation of the MS^2^ spectra (“Structural library matching based on extracted information from MS2 spectra” and “Similar structure search for aiding annotation” sections), or, most recently, generating structures based on the MS^2^ spectra or matching generated structures to the MS^2^ spectra (“In silico candidate structure generation” section). However, some candidate structures are more likely than others and can be prioritized based on experimental analytical characteristics (“Complementary features for prioritizing the candidate structures” section). For this purpose, machine learning (ML) methods have recently emerged to streamline the prediction of RT, collision cross section (*CCS*) values, adduct formation, ionizability, etc., and will be scrutinized in this review. Further, we showcase some of these approaches in the example of structural annotation of LC/HRMS features detected in a wastewater sample (“Experimental structural annotation of LC/HRMS features from a wastewater sample” section) and conclude by providing insights into future developments (“Future perspective” section). We encourage the readers to see previously published reviews on implementing analytical methods for NTS [3], chemical space detectable by NTS [4, 5], data processing and quality control workflows for NTS [6, 7], structural annotation from the metabolomics perspective [8, 9], and tools for quantification and toxicity assessment of detected chemicals [10] for a comprehensive overview of the field.

## Structural annotation based on MS^1^ and MS^2^ data

### Library MS^2^ spectra matching

**Fig. 2:**
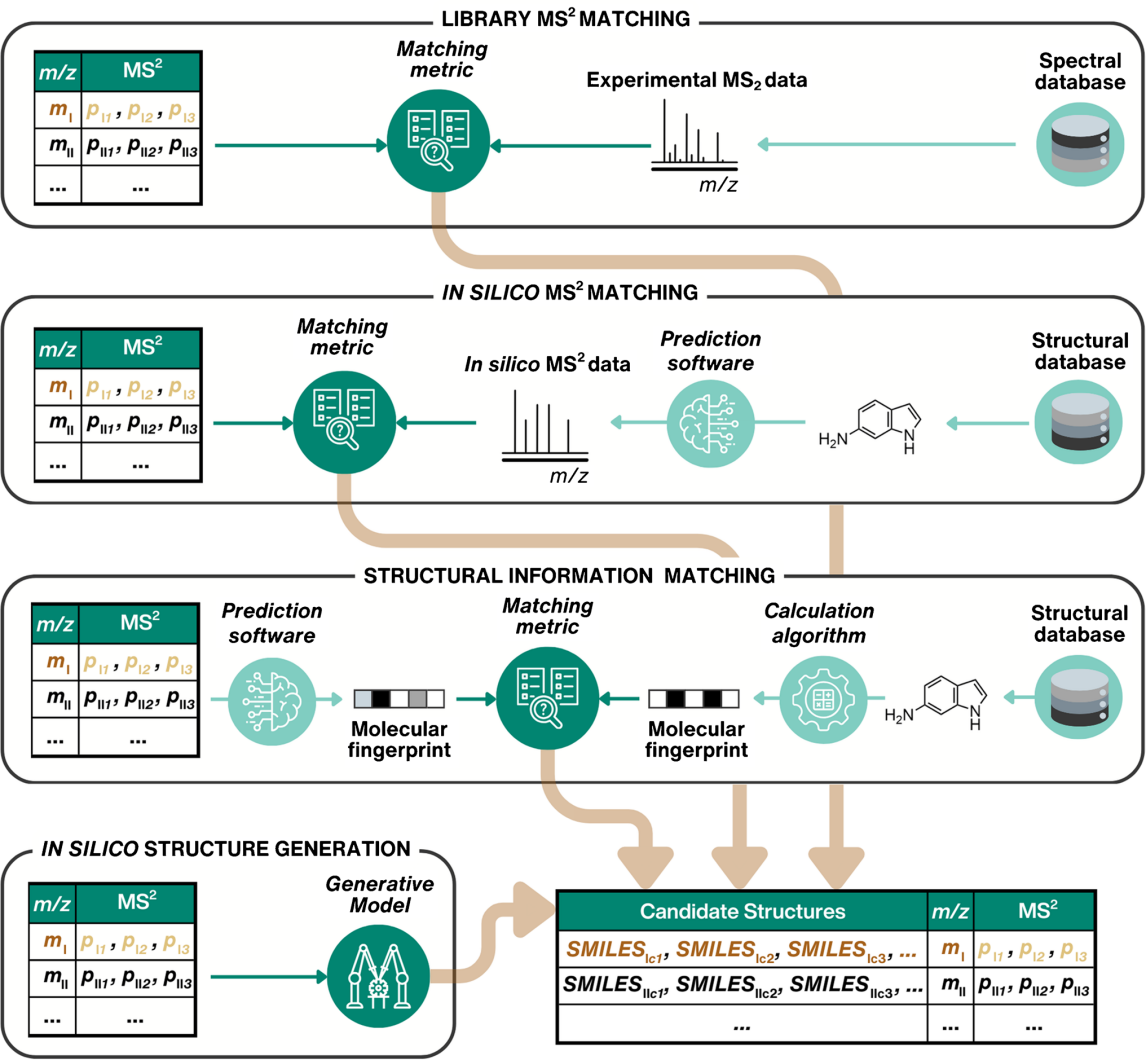
In silico approaches for retrieving candidate structures, depicted as SMILES (simplified molecular input line entry system) notations, from MS^2^ spectra. The shown MS^2^ data are arbitrarily generated and do not correspond to any specific LC/HRMS feature or structure. Brown arrows indicate that candidate structures for the same LC/HRMS feature can be obtained with all four approaches. Circled icons represent in silico components for structural annotation and prioritization; examples of these are shown in Table 1. Dark green highlights the major step of each approach, and all icons are used consistently in the following figures

Community and commercial efforts to assemble libraries of experimental tandem mass spectra enable tentative annotation of the chemicals with the highest confidence (Fig. 2), corresponding to level 2b according to the scale suggested by Schymanski et al. [11]. Some widely used libraries include MassBank Europe (MassBank) [12], MassBank of North America (MoNA) [13], National Institute of Standards and Technology (NIST) [14], METLIN [15], and Global Natural Product Social Molecular Networking (GNPS) [16].

**Table 1:** Overview of in silico components (models, algorithms, databases, metrics, and software) discussed in this review and schematically highlighted in Figs. 2, 4, and 5

In silico tool	Examples	Explanation
Spectral MS^2^ database	MassBank [12], MoNA [13], NIST [14], METLIN [15], GNPS [16]	Database containing experimental MS^2^ spectra for chemicals measured at different conditions
Spectral matching metric	Cosine similarity [17], Spectral entropy [18], MS2DeepScore [19]	Mathematical metric used to compare the similarity between two MS^2^ spectra
Software to predict MS^2^ spectra	MetFrag [20], CFM-ID [21], GrAFF-MS [22]	Algorithm or ML model predicting MS^2^ spectra for a given chemical structure
Structural database	ZINC, PubChemLite [23], NORMAN SusDat [24]	Database containing chemical structures
Software to predict structural information	BUDDY [25], SIRIUS+CSI:FingerID [26], MIST [27]	Algorithm or ML model predicting structural information such as sum formula or molecular fingerprints from experimental MS^2^ spectra
Structural matching metric	Tanimoto similarity	Mathematical metric used to compare the similarity of two structures based on their structural fingerprints
Generative model	Mass2SMILES [28], JTVAE [29], Spec2Mol [30], MassGenie [31], MS2Mol [32], MSNovelist [33],	ML model for generating chemical structures corresponding to experimental MS^2^ spectra
Empirical analytical information prediction	RTI [34], CCSbase [35], AllCCS [36]	ML model to predict a chemical property for an input structure
Empirical analytical information database	METLIN SMRT [37], RepoRT [38], UJI *CCS* Library [39], Unified CCS Compendium [40], METLIN-CCS [41]	Database containing experimental empirical analytical information of chemicals

Nevertheless, the efficiency and accuracy of the spectral library matching depend on several factors. Typically, the spectra of the unknown and reference are measured in different laboratories on different instruments with different experimental conditions. Most notably, the collision energy applied for the fragmentation of parent ions affects the extent of fragmentation [42]. Furthermore, recent studies reveal that the mobile phase composition may affect the structure of the parent ion and, consequently, the formed fragments [43, 44]. Moreover, spectral quality [45, 46] and the number of LC/HRMS features for which the MS^2^ spectra are acquired [45, 47] depend on the data acquisition method. In addition to experimental considerations, the choice of similarity metric significantly influences spectral matching (Fig. 2) [18, 48].

While cosine similarity [17] is commonly used, it may yield high similarity scores even if only one fragment matches; hence, alternative metrics, such as spectral entropy [18] or MS2DeepScore [19], have been suggested. For the spectral entropy approach, the entropy difference between the two MS^2^ spectra is calculated, whereas MS2DeepScore uses two identical neural networks to predict the structural similarity of unknown and reference chemicals as Tanimoto similarity from the respective MS^2^ spectra.

**Fig. 3:**
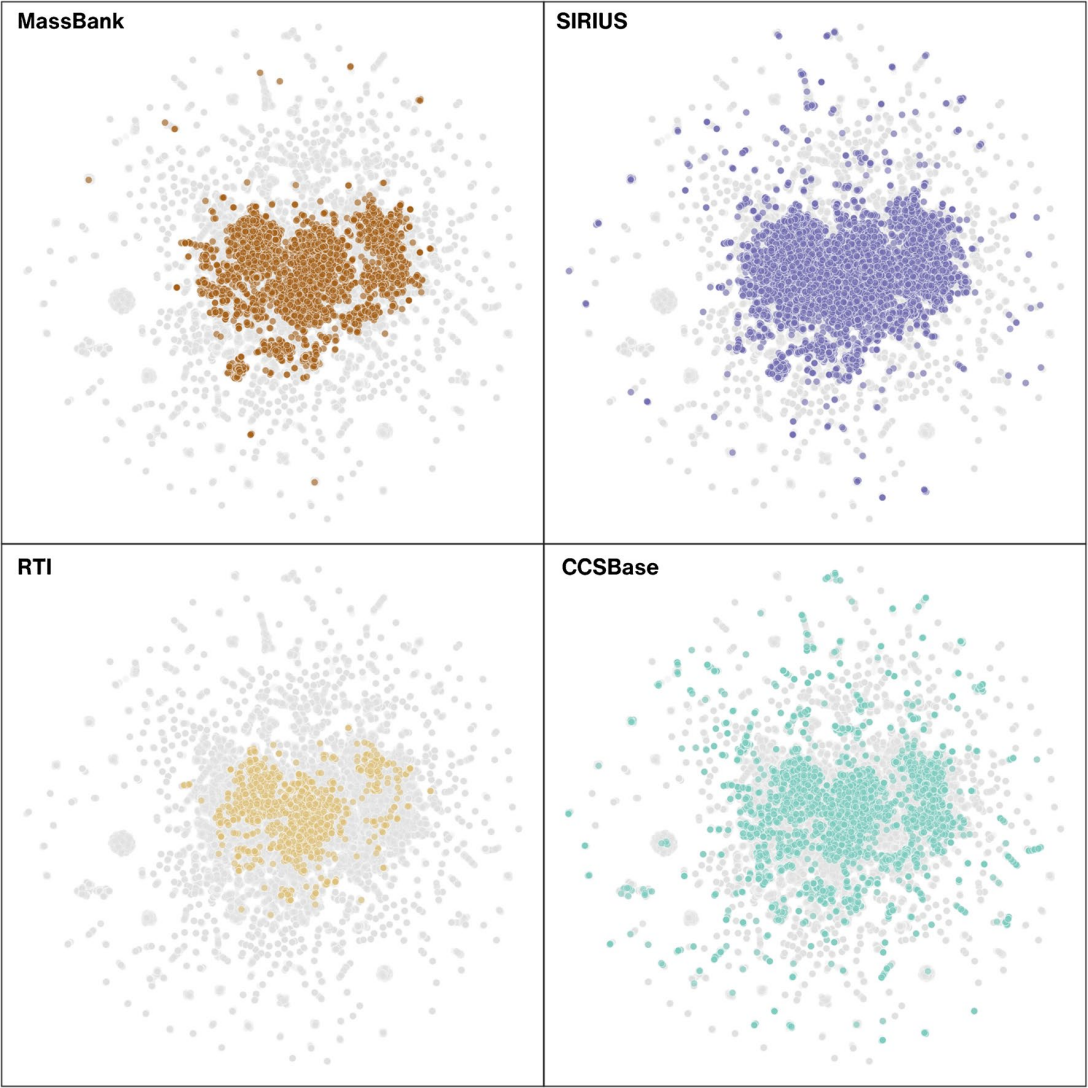
Uniform Manifold Approximation and Projection (UMAP) plots illustrating the chemical space coverage of datasets widely used for LC/HRMS feature annotation (MassBank [12] and SIRIUS [26]) and for training ML models (RTI [34] and CCSBase v1.2 [35]) applied for predicting empirical analytical information used to prioritize candidate structures. The latent space of all relevant chemicals in environmental analysis was learned based on the SIRIUS+CSI:FingerID positive mode fingerprint (3878 bits) calculated from the SMILES representation of 370,167 chemicals in the PubChemLite 0.3.0 dataset. The resulting UMAP embedding was applied to all the datasets (4310 chemicals from MassBank, 21,188 chemicals from SIRIUS+CSI:FingerID positive mode training data, 1426 chemicals from RTI training data, and 4771 chemicals from CCSBase training data). For additional details, refer to Supplementary Information 1 (SI1) Section S6

The number of experimental MS^2^ spectra ranges in thousands, e.g., MassBank 2023.11 and NIST contain reference spectra corresponding to 8215 and 47,494 chemicals, respectively, determined based on unique hashed International Chemical Identifier (InChIKeys). However, the chemicals that have not yet been measured or added to the libraries will remain unannotated. Our evaluation of the public libraries based on the 14 first characters of the InChIKey revealed that between 1.60% (MassBank) and 6.33% (NIST) of the exposure-relevant chemicals from PubChemLite [23] can be theoretically annotated with library search (Fig. 3). Thus, additional in silico approaches are needed to extend the annotations toward the remaining chemical space.

### In silico MS^2^ spectra matching

Predicting MS^2^ spectra from a known structure bridges the gap in the availability of reference MS^2^ spectra (Fig. 3). In silico library matching is based on predicting the MS^2^ spectra from the structure either by employing known fragmentation rules [49], combinatorial fragmentation [50], or competitive fragmentation modeling [51], where the latter two are widely employed by the community through MetFrag [20] and CFM-ID [21], respectively.

Despite the wide usage, in silico approaches have several known shortcomings, e.g., a large number of predicted unlikely fragments reduces the spectral similarity. Recently, Bremer et al. [52] evaluated the performance of CFM-ID 4.0 on spectra from NIST2020 and MoNA. The average dot product was below 700 out of a maximum of 1000 for most chemical classes containing heteroatoms, though the performance improved at higher collision energies where more fragments are observed. However, fine-tuning generic models for a specific class of chemicals with transfer learning has been shown to improve prediction accuracy vastly [53].

Deep learning models have accelerated acquiring fragmentation rules from the fragmentation spectra of known chemicals. For example, Young et al. [54] proposed graph transformers to predict the MS^2^ spectra, yet this approach predicts binned spectra, leading to a loss of resolution achieved by employing HRMS. To address this limitation, Murphy et al. [22] developed GrAFF-MS, where they introduced a set of molecular formulas of commonly observed fragments and neutral losses. Subsequently, a chemical structure represented as a molecular graph is mapped to the predefined molecular formulas of fragments and neutral losses, thereby preserving the spectral resolution. The quality of the predicted spectra directly impacts the annotation rates observed in environmental LC/HRMS analysis. For instance, Albergamo et al. [55] tentatively identified 884 and 550 of the 3764 and 3845 prioritized LC/HRMS features in a riverbank filtration system with MetFrag in positive and negative electrospray ionization (ESI) modes, respectively. Nevertheless, only 106 and 139 of the annotations yielded high annotation scores and were considered for further verification. Analytical standards of 42 candidate structures were tested, leading to the confirmation of 25 tentative annotations.

### Structural library matching based on extracted information from MS^2^ spectra

In an alternative approach to in silico fragmentation, the molecular formula and structural information of the candidates can be extracted from structural libraries based on the MS^2^ spectra (Fig. 2). For instance, BUDDY [25] facilitates molecular formula annotation, while SIRIUS+CSI:FingerID [26] and Metabolite Inference with Spectrum Transformer (MIST) [27] additionally provide structural annotation.

BUDDY uses the fragment and neutral loss pair information from MS^2^ for formula annotation, focusing on biochemically feasible formulas while allowing the prediction of molecular formulas beyond currently known chemicals. BUDDY annotated 193 and 53 molecular formulas absent from PubChem in plasma and urine samples*,* respectively, outperforming SIRIUS top 1 formula annotation in all tested datasets. SIRIUS [26] suggests formulas by considering mathematically plausible formulas based on the precursor’s *m/z*, isotope pattern information from MS^1^, and fragmentation from MS^2^. In the process, SIRIUS creates fragmentation trees [56] associating the formulas of fragment ions and neutral losses from MS^2^ spectra. To enable structural annotation, CSI:FingerID further predicts probabilistic molecular fingerprints based on fragmentation trees (see chemical space coverage for the training set in Fig. 3). Such fingerprints can be matched to a database of chemical structures, expanding the searchable space, for instance, to 100 million chemicals from PubChem.

Recently, MIST, a CSI:FingerID-inspired molecular fingerprint prediction tool, has been developed. Unlike other methods that rely on expert knowledge, MIST utilizes a deep learning approach and incorporates domain knowledge into its architecture. While the initial publication [27] acknowledges a limitation regarding the dependency of correct structure annotation on accurate chemical formula assignment, the subsequently introduced MIST-CF [57] improved formula annotation. MIST-CF uses an energy-based modeling framework and offers shorter calculation times than CSI:FingerID [58], which becomes important while annotating chemicals with *m/z* above 800 Da, where SIRIUS calculation can become prohibitively long. SIRIUS [26] has a graphical user interface, while MIST [27] is an open-source Python package. In metabolite annotation, MIST yielded results comparable to SIRIUS; however, its application and evaluation in environmental monitoring remain to be investigated, whereas SIRIUS has already gained widespread usage [59, 60].

### Similar structure search for aiding annotation

The end goal of environmental monitoring is to identify risk-posing chemicals; however, if the structural annotation is unfeasible due to a lack of candidate structures in databases, prioritization strategies based on chemical class or structural similarity to known chemicals can be employed. This approach is particularly valuable for annotating metabolites and transformation products, which are often absent from libraries but can be identified by retrieving structures similar to the detected chemicals [61]. Firstly, molecular networks enable linking chemicals based on their MS^2^ spectra [62]. The GNPS [16] platform facilitates this by connecting MS^2^ spectra of unknown LC/HRMS features to annotated MS^2^ spectra from all the samples available on the platform. Zhou et al. [63] increased the efficiency of structural annotation for metabolites by appending molecular networks with known reactions. This approach holds promise for annotating chemicals formed through known environmental transformations, but its application in environmental monitoring requires further exploration.

Secondly, MS2Query [64] enables the search for analog structures by matching experimental MS^2^ data with library MS^2^ spectra of similar structures using MS2DeepScore [19]. Similarly, Qemistree [65] utilizes molecular fingerprints predicted with SIRIUS+CSI:FingerID to obtain chemical trees, connecting chemicals with similar structures.

Lastly, ClassyFire [66], integrated into SIRIUS software, allows automated assignment of chemical taxonomy on up to 11 levels (Kingdom, SuperClass, Class, SubClass, etc.). Furthermore, SIRIUS enables describing detected chemicals with a CANOPUS vector, where each bit corresponds to a chemical class. These chemical classes can be used for a general description of the sample. For example, in the Earth Microbiome Project [67], ClassyFire pathway classes were used to differentiate the composition of biomes, while Sha et al. [68] tested ClassyFire’s suitability on per- and poly-fluorinated substances (PFASs) categorization, and Aurich et al. [69] grouped chemicals associated with the exposome. Spec2Class [70], on the other hand, predicts chemical superclasses directly from MS^2^ spectra, though it is currently limited to classes of plant secondary metabolites.

The approaches covered in the “Library MS2 spectra matching” to “Similar structure search for aiding annotation” sections provide numerous options to annotate LC/HRMS features with structures present in spectral or structural libraries or with structures similar to these. Nonetheless, a considerable number of LC/HRMS features in environmental samples remain unannotated [4, 32]. One of the reasons may be that novel structures, absent from libraries, remain inaccessible with these tools. We will refer to these experimental LC/HRMS features without reliable structural annotation as the “unknown chemical space.”

## In silico candidate structure generation

**Fig. 4:**
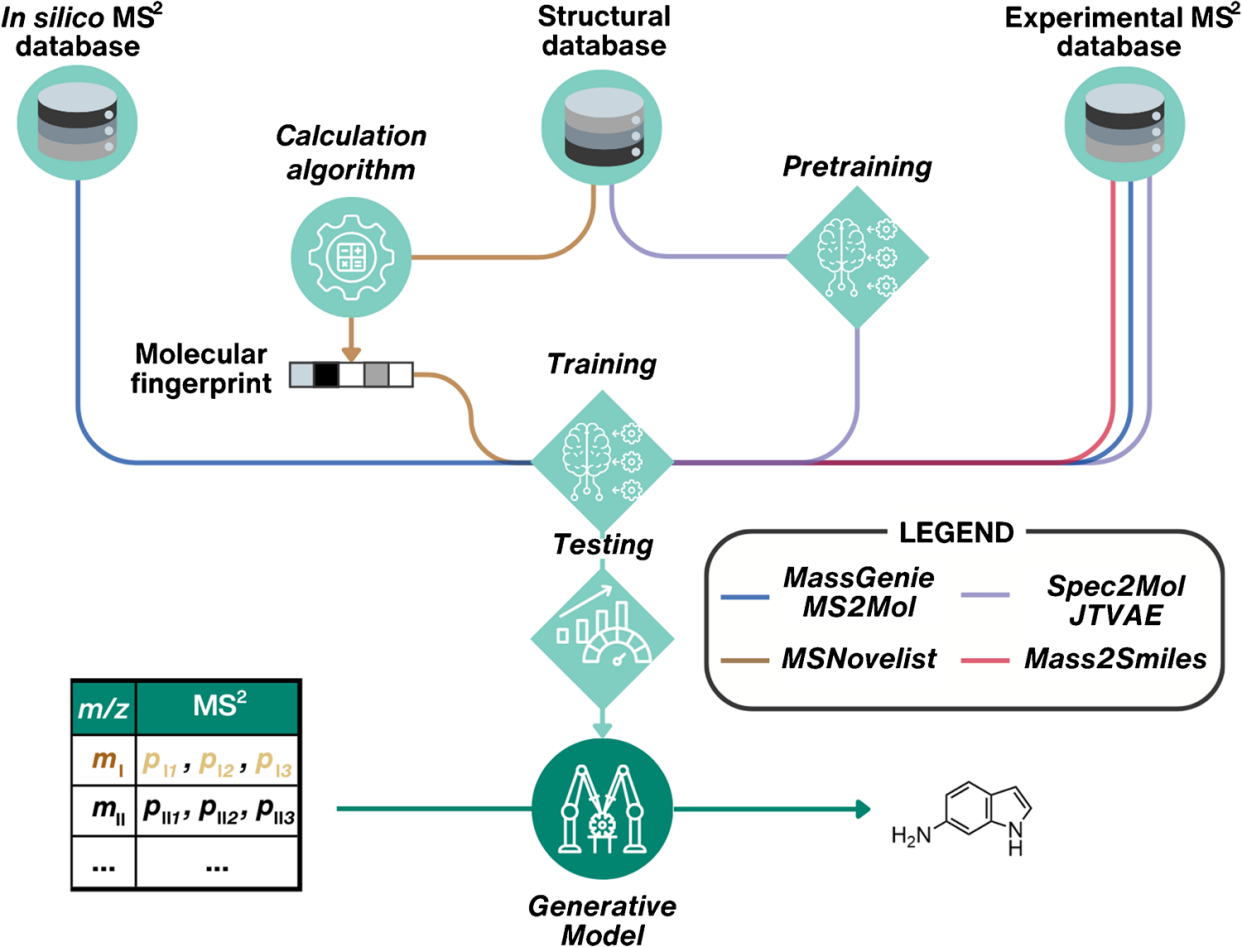
Training strategies employed by various GMs developed for candidate structure generation based on HRMS data, addressing the sparsity of training data. MassGenie [31] and MS2Mol [32] (blue) employ in silico and experimental databases for training. MSNovelist [33] (brown) is trained on the molecular fingerprints of chemicals from structural databases. The decoders of Spec2Mol [30] and JTVAE [29] (violet) are pre-trained on SMILES-to-SMILES translation. Mass2SMILES [28] (red) utilizes only experimental databases. Circled icons represent in silico components for structural annotation and prioritization; examples of these are shown in Table 1. Dark green highlights the major step of each approach, and all icons are used consistently throughout the figures of the manuscript

Exploring the unknown chemical space became recently possible with the advent of generative models (GMs) in chemistry [71]. Since 2018, GMs have leveraged deep neural networks to learn the structure-to-property distribution of large chemical datasets [72]. By treating molecular representations, such as SMILES, as a language, ML concepts from natural language processing can be adapted for property-guided in silico structure generation [71, 72]. Deep GMs, including recurrent neural networks (RNN), variational autoencoders (VAE), or transformers, have the potential to incorporate empirical analytical information from observed LC/HRMS features to guide candidate structure generation. The main obstacle to successfully employing deep GMs is the need for a sufficiently large training dataset. Usually, over 500,000 unique chemicals are considered necessary for training [33, 73], far exceeding the data available in MS^2^ repositories. Next, the recent proof-of-concept studies that have explored approaches to circumvent this problem will be discussed (Fig. 4).

Darkchem [74] represents the pioneering effort in utilizing deep GM to generate structures from LC/HRMS features. Developed by the Renslow group, Darkchem employs a VAE that takes *m*/*z* and *CCS* values as input. Transfer learning techniques were applied to address the sparse training data issue, enabling the GM to be utilized for analog search. While this method does not produce entirely novel structures and does not consider MS^2^ data, it allows the generation of structurally similar chemicals with specified properties.

In general, deep GMs trained directly on experimental MS^2^ spectra have shown limited success on validation datasets (Fig. 4) [28–30]. For instance, Mass2SMILES [28], a transformer GM, correctly annotated 1% of validation structures and yielded structures with a Tanimoto similarity score above 0.9 for 2% of cases. Spec2Mol [30] consists of a decoder trained in SMILES-to-SMILES translation on 135 million chemicals from PubChem and ZINC-12, coupled with an encoder trained on MS^2^ spectra for over 30,000 chemicals from NIST2020 (Fig. 4). On the CASMI2017 challenge dataset, Spec2Mol yielded correct structures for 7% of MS^2^ spectra, compared to 67% for SIRIUS [26]. However, in cases where SIRIUS provided incorrect structures, on average, Spec2Mol suggested candidate structures with higher fingerprint similarity to the correct structure.

Data sparsity can be overcome by training GMs on experimental and in silico generated MS^2^ spectra (Fig. 4), as demonstrated in MassGenie [31]. For training, it exploits in silico binned MS^2^ of chemicals from the ZINC library generated by a method based on MetFrag [23] and correctly predicted 53% of the structures with *m/z* below 500 Da in the CASMI2017 challenge dataset. MS2Mol [32] employs a similar approach, utilizing a transformer trained on experimental spectra for 50,083 chemicals, augmented with in silico spectra predicted with CFM-ID [21] for chemicals of the LOTUS dataset. Notably, MS2Mol uses tokenized MS^2^ spectra as input, retaining high-resolution information from MS^2^ compared to binned spectra. Although CSI:FingerID outperformed MS2Mol on established datasets such as CASMI2022, evaluating GMs solely on known chemical space does not adequately test their ability to generate unknown structures and leads to the overestimation of the performance of the models trained on familiar data. To mitigate this, the authors of MS2Mol evaluated its performance on an EnvedaDark dataset of unknown structures. MS2Mol (21.4%) outperformed both CSI:FingerID (11.0%) and library search (7.2%) on close matches (Tanimoto similarity > 0.675). These findings highlight the challenges of providing a suitable validation set for GMs.

By leveraging molecular fingerprints calculated based on MS^2^ spectra with CSI:FingerID [58], MSNovelist assembled these fingerprints to candidate structures using an RNN [33]. This workaround enabled training the MSNovelist on 1,000,000 fingerprints of known structures without the need for MS^2^ spectra matching (Fig. 4). However, this method’s effectiveness depends on the quality of the fingerprints obtained with CSI:FingerID. While MSNovelist generated 45% correct structures for a GNPS-based test set compared to 75% with fingerprint database matching, it proposed new metabolites for a bryophyte dataset, outperforming the best database candidates.

Employing an RNN, DarkNPS adopts a more targeted approach to construct a structural library of novel psychoactive chemicals [53, 75]. The GM is trained to generate structures similar to known psychoactive chemicals, and the outputted structures are prioritized based on experimentally observed MS^1^ and MS^2^, leveraging in silico MS^2^ spectra generated with CFM-ID [21]. However, no mass spectrometric data is involved in the model training. This method exhibits promising results within a suspected chemical space, yet limitations remain in its applicability for unknown chemical space.

Due to the novelty of the field and limited code availability, applying GMs to environmental samples remains unexplored. Additionally, all deep GMs described require substantial computational resources for training, though employing pre-trained models for structure generation requires less computational effort. Furthermore, evaluating the performance of GMs is challenging, necessitating benchmarking datasets of unknown structures and posing difficulties in comparing methods due to differing applicability domains.

## Complementary features for prioritizing the candidate structures

The methods discussed earlier utilize MS^1^ and MS^2^ data to obtain a candidate list. Consequently, complementary experimental information is essential to deprioritize unlikely and prioritize more plausible candidate structures. Empirical analytical information, such as RT from LC and *CCS* from IM measurements, are commonly used for this purpose. Moreover, insights into ion formation via ESI and sample-related details prove invaluable. While this information can serve as expert knowledge for prioritization, such as considering seasonal disease risks to identify potential chemicals in municipal wastewater [76] and thereby selecting more probable candidates, relying solely on this approach is time-consuming and fraught with challenges. Therefore, in silico methods for removing false positives from the candidate list have emerged.

**Fig. 5:**
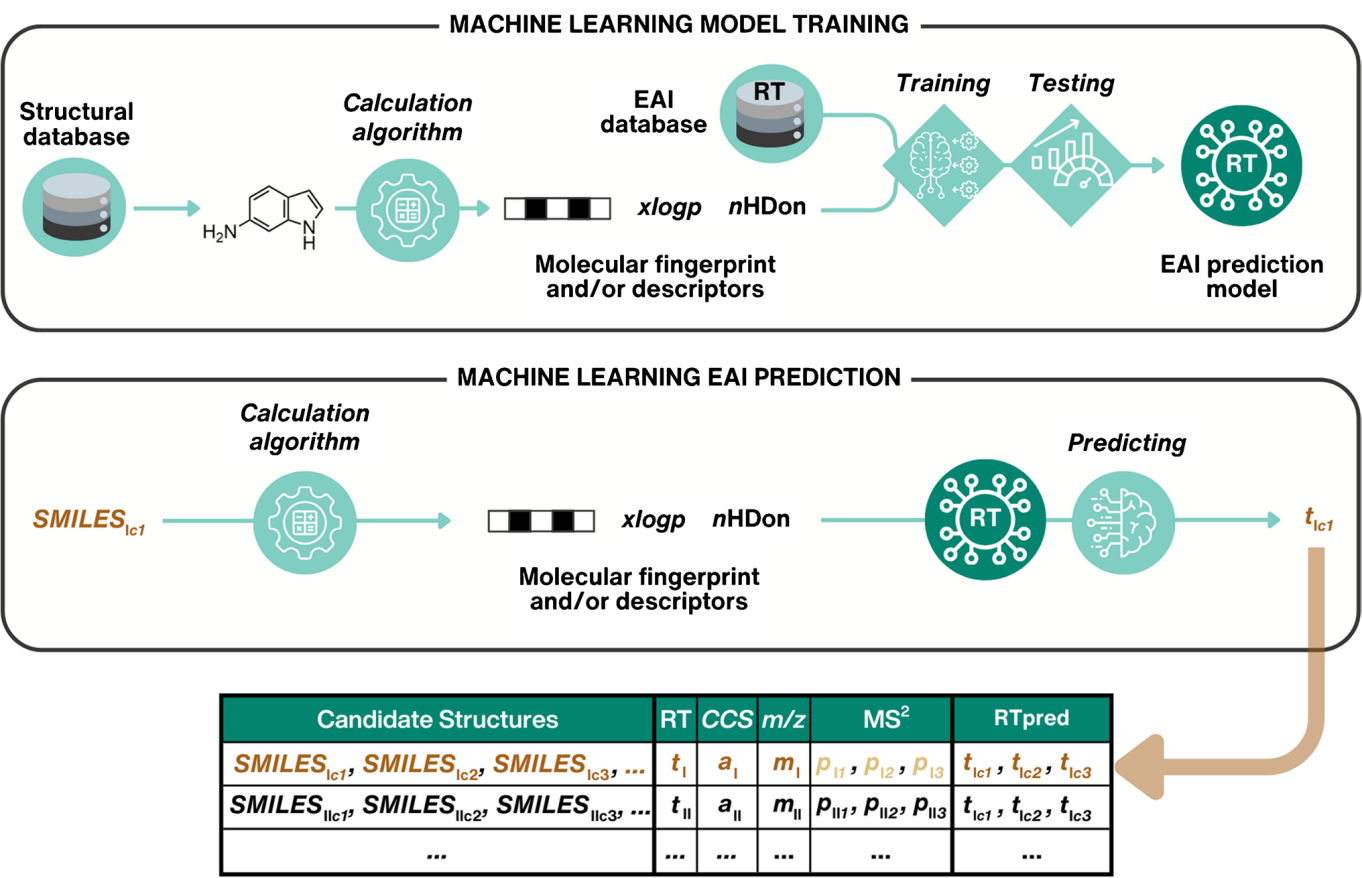
Computational workflow illustrating the training process of an empirical analytical information (EAI) prediction model using RT as an example. The model is trained by utilizing molecular fingerprints and/or descriptors, followed by empirical analytical information prediction for candidate structures. The brown arrow indicates that retention times can be predicted for each candidate structure. Circled icons represent in silico components for structural annotation and prioritization; examples of these are shown in Table 1. Dark green highlights the major step of each approach, and all icons are used consistently throughout the figures of the manuscript

These methods operate under the assumption that the complementary information available for prioritization is determined by the structure of the chemical and, consequently, its physicochemical properties. For example, the carbon chain length of PFASs affects the RT [77]; thus, the relationship between RTs and structures of PFASs can provide support for identification within a group of homologs. In silico techniques, including ML models, are developed to uncover the relationships between structural and empirical information (Fig. 5). Afterward, these models are employed to predict empirical analytical properties for each candidate structure, and poor agreement with experimental data suggests lower plausibility of the candidate structures.

The potential of in silico approaches was demonstrated by Song et al. [78], who developed an RT prediction model for contaminants of emerging concern and applied it in NTS of wastewater. The group annotated 719 LC/HRMS features with varying confidence levels through comparison with reference standards and matched the obtained MS^2^ spectra with experimental and in silico MS^2^ spectra from libraries. Subsequently, the developed RT model was employed to improve the confidence of 234 LC/HRMS features. The annotation confidence of 153 out of these features could be enhanced by leveraging the predicted RT values. In another study by Bijlsma et al. [79], the number of candidate structures was reduced by between 43 and 66% by applying predicted RT and *CCS* values. In the following sections, we will delve into the in silico methods and provide details of their applicability, potential, and practical implementation in NTS workflows.

### Leveraging retention time in NTS workflows

RT stands as the most prevalent complementary information employed within NTS workflows. This is because even slight structural differences can result in notable variations in RT, aiding in the differentiation between isobaric and isomeric chemicals. A straightforward approach would involve comparing the RT obtained experimentally via LC/HRMS with the RT of candidate structures stored in databases. One of the most widely known and largest datasets of RT values is the METLIN small molecule retention time (SMRT) dataset [37], comprising information about 80,038 small molecules analyzed on reversed-phase LC. However, this approach encounters challenges due to the wide variety of chromatographic conditions (column, mobile phase, gradient, etc.) used, making it practically impossible to obtain exhaustive RT libraries. Consequently, in silico models have been developed to predict RTs.

These models utilize chromatographic conditions [80], physicochemical properties [81], molecular descriptors [82–84], molecular fingerprints [37], or their combination [85] as input and leverage various ML approaches, such as generalized additive models [80, 86], multiple linear regression, support vector machines, random forest, gradient boosting, and artificial neural networks [87–92]. However, their applicability is often hindered by the variability in RTs across different chromatographic systems. To mitigate this challenge, models have been developed to project known RTs from one chromatographic condition onto another [93]. Another hurdle in developing robust and generalizable models is the scarcity of experimental datasets required for model training and the insufficient overlap of the chemical space between the training data and the applicability domain. For instance, the RT models trained on metabolites will likely be less accurate for PFASs. To address this issue, the RepoRT [38] data repository has been recently established. This extensive metadata-rich collection comprises 373 datasets containing 88,325 RT values for 8809 unique chemicals measured using 49 different chromatographic columns, in addition to the METLIN SMRT dataset. Moreover, innovative methods integrating deep learning and transfer learning have emerged to tackle the constraints of RT models to specific chromatographic conditions and the availability of pertinent data [94–98].

Besides the models that directly predict the RT values, the retention time index (RTI) system [34], with RTI values ranging from 1 to 1000 and based on the elution patterns of calibrants, has been proposed to harmonize RTs across LC studies. Unlike gas chromatography, where RTI usage is well-established, its adoption in LC has been hindered by the diverse range of chemicals and properties affecting separation [34]. However, the external validation of the RTI system and its accessibility through the University of Athens platform (http://rti.chem.uoa.gr/) for RTI calculations, coupled with insights into uncertainty and applicability domain (see Fig. 3 for coverage of chemical space), have gained considerable recognition within the scientific community.

Despite significant advancements and the development of hundreds of models for predicting RTs of small molecules, their accuracy remains imperfect. Moreover, there are cases where accurate RT prediction is particularly challenging, especially in the presence of what we term “RT cliffs”, where molecules with very similar structures exhibit notably different RTs (e.g., *cis*- and *trans*-isomers). Since models rely on structural information, capturing subtle structural variations that influence RTs can be exceedingly difficult, if not impossible, using conventional input data such as fingerprints or molecular descriptors. This highlights the ongoing need for research and innovation to enhance the accuracy and reliability of RT prediction models, particularly in complex chromatographic systems.

### Leveraging ion mobility in NTS workflows

IM separates ions based on size, shape, and charge as they travel through a buffer gas in an electric field [99]. This provides an additional dimension of separation and allows deconvoluting MS^2^ spectra in data-independent acquisition (DIA) where coeluting chemicals within a specific *m/z* range would otherwise be fragmented together, leading to complex MS^2^ spectra [100]. Furthermore, it can facilitate the separation of isomeric and isobaric chemicals [101], and *CCS* values, derived from the drift time of the chemical, can increase structural annotation confidence. The latter can be achieved by matching experimental *CCS* values with either known *CCS* values of candidate structures from databases or with predicted values [39]. For library matching, databases such as the UJI *CCS* Library [39], the Unified CCS Compendium [40], and the most recent METLIN-CCS [41] contain *CCS* values for over 27,000 structures.

Predicted *CCS* values can be obtained through computational modeling or ML techniques. Modeling relies on computing the lowest energy conformer of the detected ions followed by *CCS* determination with different algorithms [102], such as IMoS or MobCal [103]. Conversely, ML approaches, such as CCSbase [35] and AllCCS [36], rely on molecular descriptors [36, 83, 92]. However, including *m/z* as one of the most significant descriptors in these models reduces their orthogonality to MS^1^ [36], leading to varying levels of success in NTS applications. Although the efficiency largely depends on the LC/HRMS feature, Asef et al. [104] found that applying predicted *CCS* values reduced the candidate list by an average of 28%. The low efficiency in deprioritizing candidate structures is mainly attributed to the accuracy of predicted and measured *CCS* values. Typically, predicted and experimental *CCS* values are considered to match within a difference of 3% [104, 105], while the measured *CCS* values of isomeric chemicals can differ by less than 1% [106]. This is also highlighted by Akhlaqi et al. [107], who found that the confidence interval of *CCS* values needs to reach 0.04–0.15% depending on the dataset to reduce the number of candidate structures by 95%. Additionally, predicted *CCS* values for structures out of the ML model’s applicability domain (see Fig. 3 for coverage of chemical space of commonly applied CCSbase as an example) may increase the uncertainty.

### Additional experimental information for prioritization in NTS

**Fig. 6:**
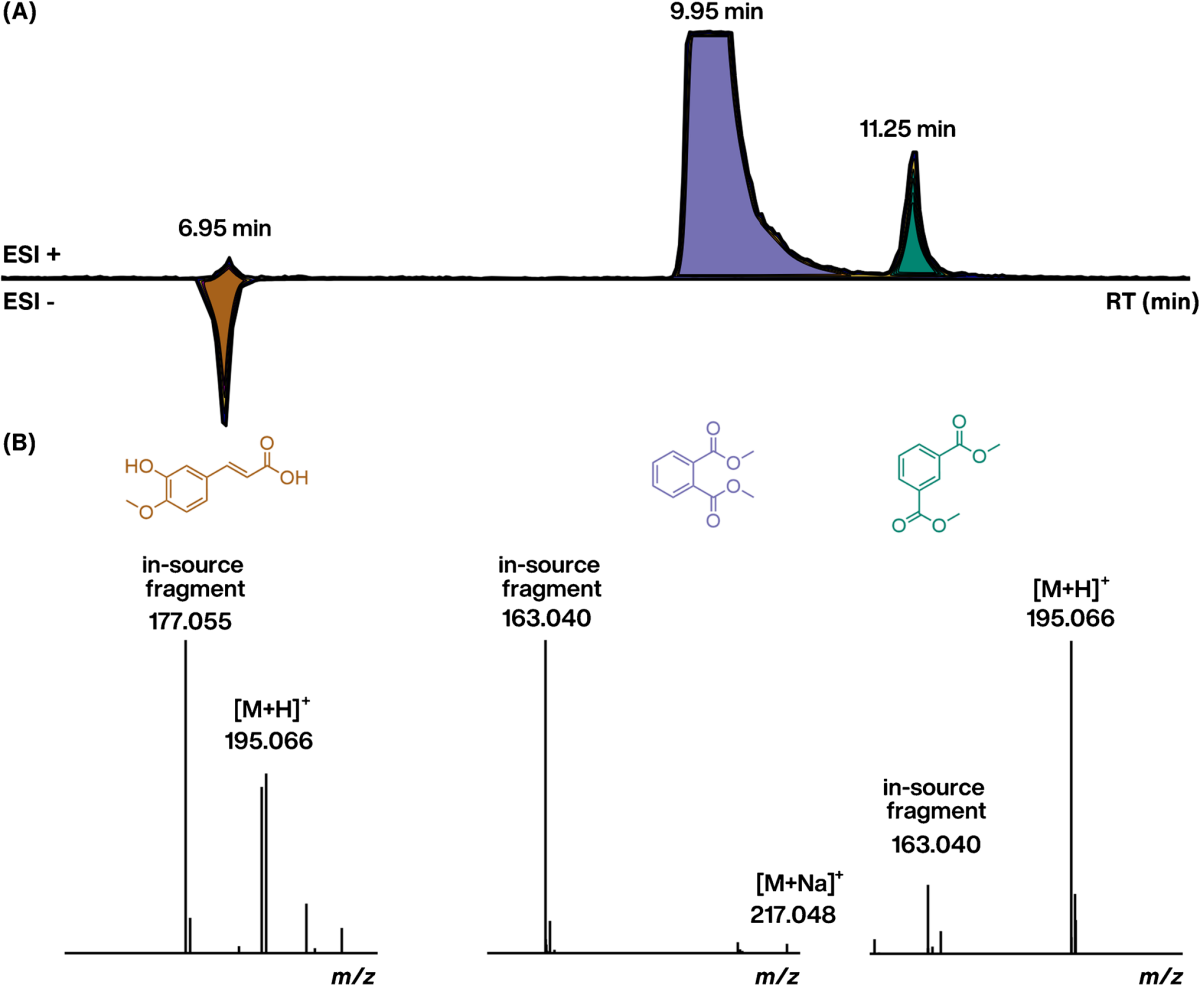
Three chemicals sharing the same molecular formula (C_10_H_10_O_4_) can exhibit distinct retention times and be detectable with different ESI modes, influenced by their polarity and acid–base properties. **A** The peak corresponding to dimethyl phthalate (violet) is magnified by a factor of 10 × for enhanced visibility of other chromatographic peaks. **B** Additionally, adduct formation and in-source fragmentation may offer supplementary insights into the localization of functional groups

Although RT and *CCS* values are already used for evaluating candidate structures, confidence can be increased by considering further analytical information. For instance, the ion type observed in ESI/HRMS spectra can be highly indicative of the functional groups and their positions in the detected chemicals (for an experimental case study, see Fig. 6). For example, deprotonated molecules are observed in ESI negative mode for chemicals with acidic functionality, whereas protonated molecules are observed in positive mode for weak and strong bases. Thus, Lowe et al. [108] proposed a concept of ESI/HRMS amenability leveraging the probability of observing [M+H]^+^ or [M−H]^−^ for specific candidate structures. Our group has shown that the ionization efficiency of both ion types can be predicted from the chemical structure [109], suggesting the possibility of using the relative response in these two modes.

Moreover, sodium, potassium, and ammonium adducts are frequently observed in ESI positive mode, and the formation probability of specific adducts may become beneficial in the structural annotation. Our group [110], as well as Broeckling et al. [111], have investigated the possibility of predicting adduct formation through classification and regression, respectively. Additionally, physiochemical properties, e.g., equilibrium partition ratios between organic solvents and water, have been utilized to differentiate isomers. Abrahamsson et al. [112] used peak intensities in water and eight different organic solvent systems to predict molecular fingerprints, enabling database searches for chemical structure.

In addition to analytical techniques commonly coupled to HRMS, spectroscopic techniques, such as gas phase infrared spectroscopy, can provide complementary information and improve the annotation accuracy [113]. In combination with MS^2^ and domain knowledge, this technique has recently enabled the identification of organic micropollutants in wastewater [114]. Nevertheless, access to such complementary tools is limited to non-standard instrumentation.

## Experimental structural annotation of LC/HRMS features from a wastewater sample

**Fig. 7:**
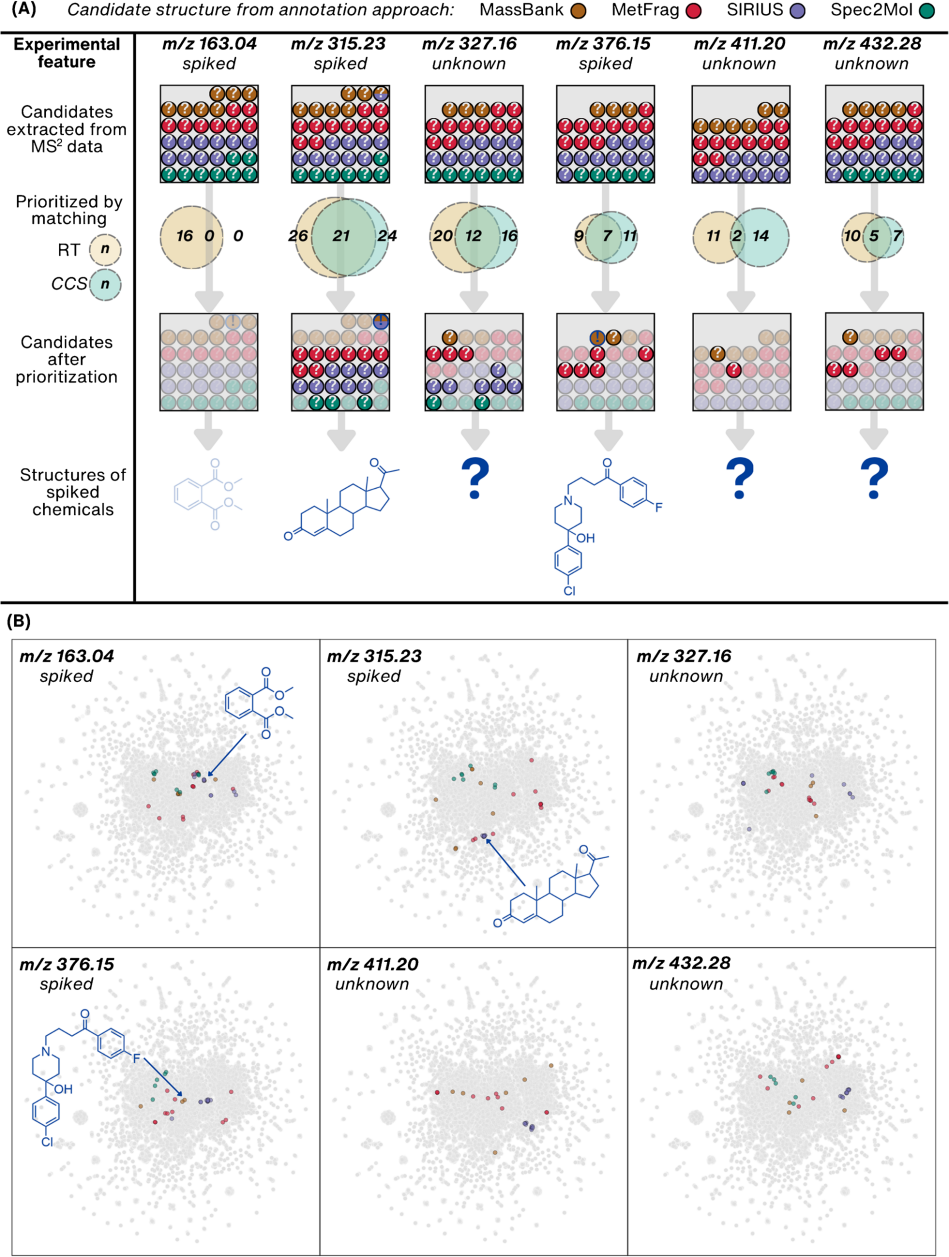
Visualization of the structural annotation and candidate structure prioritization results for the six LC/HRMS features out of the 20 LC/HRMS features studied (remaining features are provided in the S11 Section S8). **A** The number of candidate structures obtained from experimental and in silico spectra matching with MassBank and MetFrag, and by employing SIRIUS+CSI:FingerID and Spec2Mol. Each candidate structure is represented by a colored circle, with the order indicating its rank within the annotation approach. Dual-colored circles represent candidate structures suggested by two methods. The middle panel illustrates the number of candidate structures prioritized based on predicted RT obtained by utilizing the RTI model and *CCS* obtained by employing the CCSbase model. For features corresponding to the spiked chemicals, the correct structure is highlighted with a dark blue exclamation mark. **B** Visualization of the candidate structures in the chemical space using the UMAP embedding of PubChemLite (Fig. 3). All points are transparent, resulting in a darker color when data points are overlaid

To illustrate the NTS workflows, we investigated structural annotation and prioritization of 10 spiked chemicals treated as unknowns, alongside 10 truly unknown experimental LC/HRMS features from the wastewater sample. We conducted experimental and in silico spectra matching with MassBank [12] and MetFrag [20], as well as employed SIRIUS+CSI:FingerID [26] and Spec2Mol [30] to retrieve candidate structures from MS^2^ data (for experimental and data processing details, see SI1 Sections S2–S4). We considered the top 10 candidate structures per LC/HRMS feature from each of the four methods, excluding the chemically invalid structures, resulting in 515 unique candidate structures, with 253 for spiked chemicals and 262 for truly unknown features. The candidates obtained for three randomly selected spiked and three randomly selected unknown LC/HRMS features and their following prioritization are visualized schematically in Fig. 7A (for other features, see SI1 Section S8). Additionally, the structures of all the candidates can be found in the SI1 Section S9.

Employing the standard practice in evaluating the performance of NTS workflows, we first assessed the applicability and effectiveness of the methods on the annotation of spiked chemicals (Table 2). MassBank accurately annotated all spiked chemicals within the top 3 candidates, while SIRIUS+CSI:FingerID correctly annotated half of the features (always as top 1). In the other five cases, SIRIUS+CSI:FingerID also annotated the sum formulas incorrectly. For example, as seen in Fig. 7A, for the spiked feature with *m/z* 163.04, dimethyl phthalate, detected as an in-source fragment in MS^1^, was correctly annotated by matching with MassBank, but not by SIRIUS+CSI:FingerID. This underscores the advantage of parent mass independent library matching, which is becoming beneficial in all-ion fragmentation (AIF) or DIA analyses. Moreover, neither MetFrag nor Spec2Mol suggested correct structures within the top 10 candidates for any of the spiked chemicals. The underperformance of Spec2Mol may be attributed to the fact that the authors observed the best results when utilizing MS^2^ data of both positive and negative ionization modes [30], but only positive mode data was considered here. However, evaluating and comparing structural annotation methods using only spiked chemicals can lead to biased conclusions, as these chemicals originate from the known chemical space within databases and training sets used by the models/tools. Thus, we suggest evaluating NTS workflows additionally on unknown features, although, due to the absence of ground truth, this evaluation requires different approaches or additional analytical experiments, such as isolation and NMR analysis.

**Table 2:**
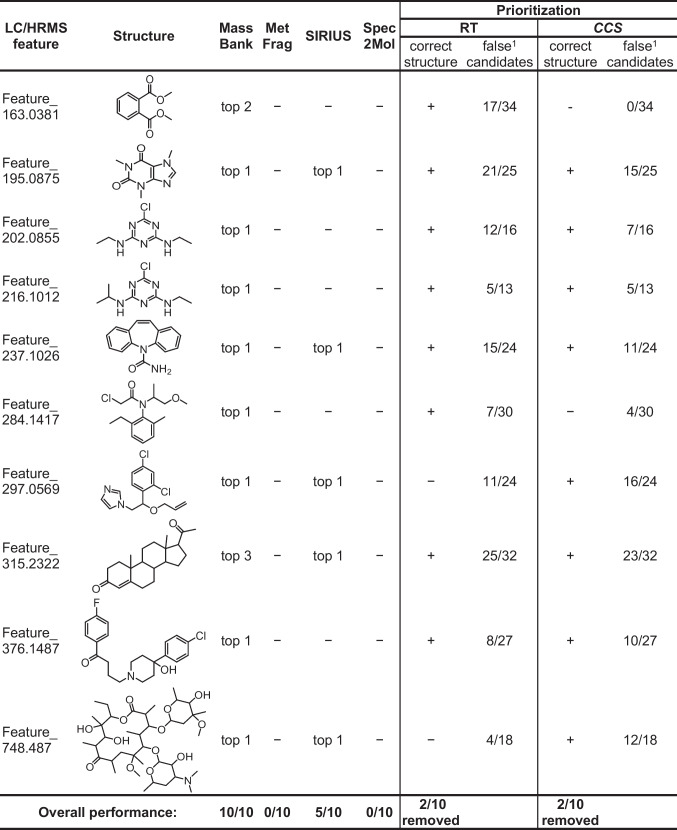
Evaluation of the structural annotation and candidate prioritization of spiked chemicals

^1^Ratio of wrong candidate structures prioritized from all false candidates for one LC/HRMS feature

**Fig. 8:**
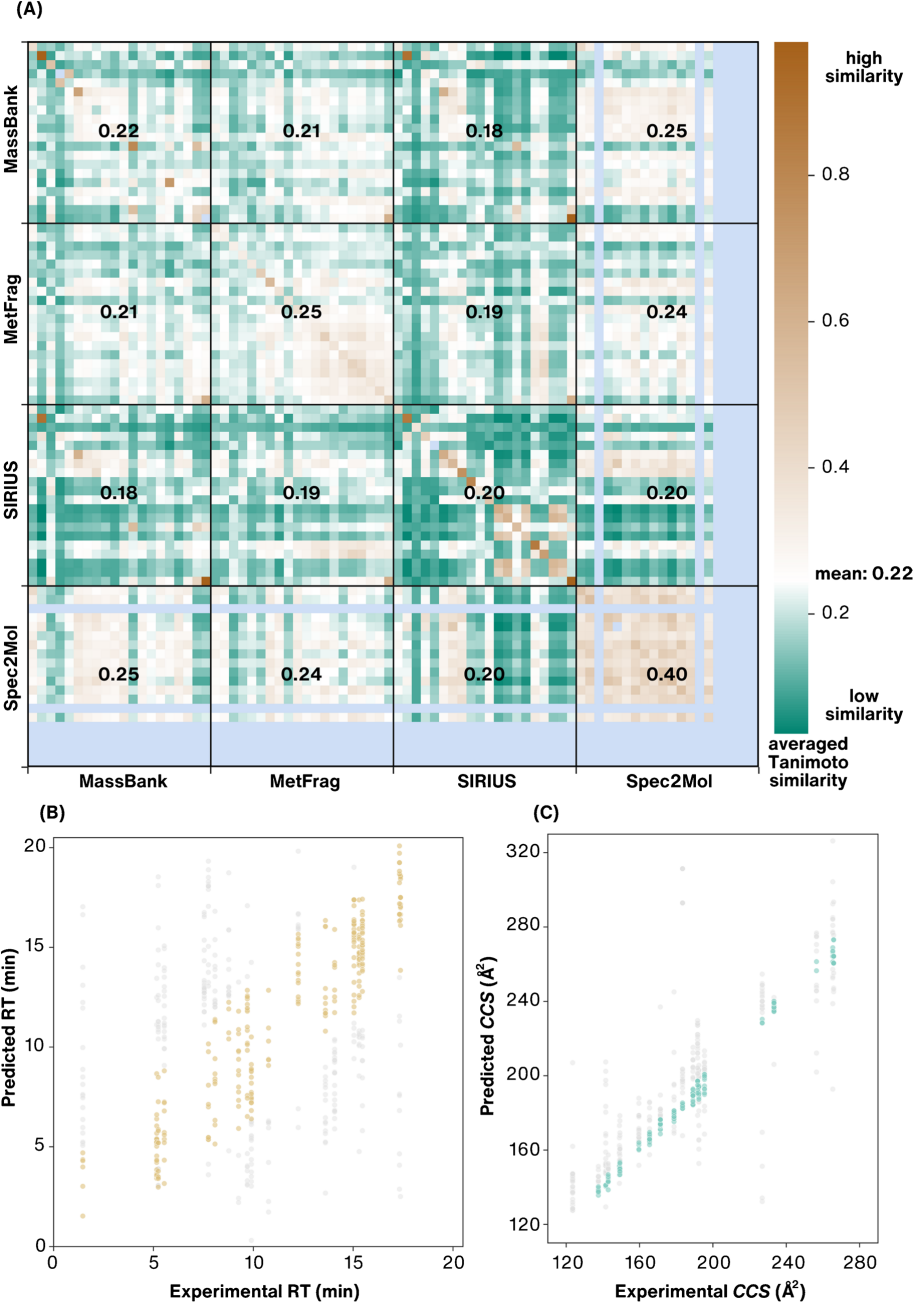
**A** Heatmap illustrating the structural similarities among candidates suggested by four methods employed for the structural annotation of 20 LC/HRMS features (SI1 Section S7). Each small colored square represents the similarity of candidate structures, calculated as the average of all pairwise Tanimoto similarities between all the suggested candidates within one LC/HRMS feature. The LC/HRMS features are sorted based on their *m/z* values. Brown indicates higher similarity, while green indicates lower similarity among candidate structures (the white midpoint of the colorbar (0.22) denotes the average similarity across all the suggested candidate structures, calculated as the mean using all the pairwise Tanimoto similarities of candidates). Light blue indicates that a specific LC/HRMS feature did not yield any candidate structures from a particular method. Numbers inside the larger squares represent the overall average similarity scores within or between the methods. **B** Experimentally obtained retention time (RT) values for 20 LC/HRMS features plotted against the predicted RT values from the RTI model for all candidate structures corresponding to each LC/HRMS feature. Candidates prioritized using the cutoff criterion of ± 2 standard residuals are highlighted in light brown. **C** Experimentally obtained *CCS* values for 20 LC/HRMS features plotted against the predicted *CCS* from the CCSbase model for all candidate structures corresponding to each LC/HRMS feature. Candidates prioritized using the criterion of difference between predicted and experimental values less than 3% are highlighted in light turquoise

Furthermore, we analyzed the candidates obtained from the four annotation tools by comparing the average Tanimoto similarity scores (Fig. 8A), unveiling that all methods propose structurally similar candidates within one LC/HRMS feature. Moreover, when scrutinizing the similarities of candidates suggested by different methods for the same feature, it becomes apparent that employing SIRIUS+CSI:FingerID and library matching with MassBank yield similar candidates. This aligns well with the observed good performance of both methods on the spiked chemicals. Candidate structures between different LC/HRMS features vary most for SIRIUS+CSI:FingerID. In contrast, the chemically valid structures generated with Spec2Mol exhibit general similarity across all the LC/HRMS features. Comparable observations can be deduced from the visualizations, where each candidate structure per individual LC/HRMS feature is mapped to the chemical space (Fig. 7B and SI1 Section S8). These findings suggest that the candidate structures generated by Spec2Mol overlap with the known chemical space, which could imply that the experimental LC/HRMS features originate from the known chemical space. Alternatively, this overlap may highlight the limitations in the applicability of current GMs, as discussed in the “In silico candidate structure generation” section.

For prioritizing candidate structures, we utilized predicted RT and *CCS* values by employing RTI [34] with the cutoff criterion of ± 2 standard residuals and CCSbase [35] with the difference between predicted and experimental *CCS* below 3% (see SI1 Section S5 for details). Out of 515 candidates, 255 (depicted in light brown in Fig. 8B) were prioritized based on RT, and 208 (depicted in light turquoise in Fig. 8C) based on *CCS* (Table 2). However, the significant number of remaining candidates underscores the inefficiency in prioritization due to the high uncertainty in RT and *CCS* prediction, highlighting the need for further advancements in in silico methods. Additionally, the RTI model was unable to predict RT for two candidate structures and CCSbase for four structures, indicating the limitations in applicability, possibly stemming from the insufficient coverage of chemical space by the data used for model training (Fig. 3). For instance, the RTI model’s inability to predict the RT for a boron-containing candidate can be attributed to the absence of boron chemicals in the training data. Applying both methods for prioritization resulted in 66 candidates for 9 spiked chemicals and 43 for 8 truly unknown LC/HRMS features. Consequently, for one spiked chemical and two truly unknown features, all candidate structures were deprioritized (Fig. 7A, SI1 Section S8). Furthermore, the previously mentioned example of a spiked feature with *m/z* 163.04, an in-source fragment of dimethyl phthalate, highlights the challenges faced by in silico methods in accurately prioritizing candidates for unknown structures observed as unusual fragments or adducts. Here, the correct structure was deprioritized due to a mismatch in *CCS* values, as the experimental *CCS* corresponded to the in-source fragment rather than the parent ion of the correct candidate structure. Similarly, the correct structures for the spiked features *m/z* 284.14, 297.06, and 748.49 were deprioritized due to discrepancies in *CCS* for the first and mismatching RT for the latter two (Table 2 and SI1 Section S8). This underscores that the high measurement and prediction uncertainty of current methods impedes reliable candidate prioritization.

## Future perspective

Despite significant advancements in in silico annotation tools in recent years, further improvements are required to increase the annotation rates, accuracy, and reproducibility. Below, we outline three main considerations that arose from compiling this review.

### Chemical space coverage

The annotation efficiency of the spectral library and in silico spectra matching is determined by the extent to which the chemical space of the employed database overlaps with the chemical space of the sample. Moreover, the accuracy of ML models hinges on the alignment of the sample’s chemical space with the models’ training set [115]. However, achieving comprehensive coverage across a vast spectrum of environmentally relevant chemicals is challenging due to variations in sample sources (e.g., water vs soil, residential area vs industrial dumping site). Additionally, empirical analytical information, such as retention time [38, 116], strongly depends on the analytical method used for NTS. Consequently, predictions often require calibration or transfer to the system used for analysis. Yet, applicability is ambiguous if the conditions differ significantly for the training data. Unfortunately, most tools do not explicitly indicate the similarity between candidate structures and training data, making it challenging to assess whether unknown or tentatively identified chemicals fall within the ML models’ applicability domain. Thus, evaluating the overlap of candidate structures’ chemical space with the training set or database can offer insights into structural annotation accuracy.

### Model evaluation

Most ML models are evaluated based on conventional training metrics, such as root mean square error for regression tasks. Nonetheless, these metrics may not precisely measure the efficiency in prioritizing candidate structures [104, 107]. Namely, candidate structures obtained from one MS^2^ spectrum often share the same chemical formula and highly similar structure. Hence, we encourage the developers to invest additional effort in customizing evaluation metrics, such as integrating uncertainty, to suit specific use cases and to incorporate them when benchmarking the models. Furthermore, curated datasets separate from those used for model training should be employed for evaluation. The latter is particularly challenging for MS^2^ annotation strategies, as most public libraries have been utilized, directly or indirectly, in developing and optimizing currently available annotation approaches. In this matter, EnvedaDark [32] is the first such library; however, careful measures are necessary to prevent the leakage of such libraries into model training.

### Combining in silico methods

Combining multiple structural annotation strategies can be advantageous, as consensus among different methods may reinforce the plausibility of a candidate structure. This was also evidenced in our experiments, where alignment between candidate structures from library matching with MassBank and SIRIUS+CSI:FingerID occurred for the correct candidate structure for spiked chemicals. Furthermore, various empirical analytical characteristics described in the “Complementary features for prioritizing the candidate structures” section can aid in prioritizing structure identification. However, there is currently a lack of community-wide strategies for managing predictions, particularly in combining predictions from different empirical analytical characteristics with varying uncertainty and accuracy. Nonetheless, the integration of such predictions is highly desirable due to the complementary nature of retention time, *CCS* values, adduct formation, and mass spectrometric information.

## Conclusion

In this critical review, we have outlined and compared methods for retrieving candidate structures to annotate unknown LC/HRMS features. As expected, spectral library matching offers the most reliable results when the sought structure is available in the library. However, the currently publicly available spectral databases cover only a fraction of the chemical space. GMs offer access to yet-unknown structures, but their effectiveness depends on the comprehensiveness of the training data. Additionally, the significance of methods leveraging complementary empirical information for candidate structure prioritization cannot be overstated. Inaccurate predictions of properties for candidate structures beyond the model’s applicability domain may result in the loss of correct candidates. Importantly, some candidate structures may be disregarded even before applying these methods if the adduct type cannot be definitively determined or if the deconvolution of data-independent tandem mass spectra produces noisy results.

## Supplementary Information

Below is the link to the electronic supplementary material.

Supplementary Information 1 (DOCX 22825 KB)

Supplementary Information 2 (CSV 122 KB)

## Data Availability

The Supplementary Information 1 includes details on experimental methods, data processing, and figures. Additionally, a table containing all extracted candidates and their prioritization can be found as Supplementary Information 2. The code for data processing and visualization is available at the GitHub repository: https://github.com/kruvelab/NTS_LC_HRMS.
