# Supplementary material for: Critical review on in silico methods for structural annotation of chemicals detected with LC/HRMS non-targeted screening
Source: Anal Bioanal Chem. 2024 Aug 14;417(3):473–93. doi: 10.1007/s00216-024-05471-x (PMC11700063; doi:10.1007/s00216-024-05471-x)
Supplement: Supplementary file 1 — Supplementary Information 1 (DOCX 22825 KB) [file 216_2024_5471_MOESM1_ESM.docx]

Supplementary Information 1 for

**Critical Review on *In Silico* Methods for Structural Annotation of Chemicals Detected with LC/HRMS Non-Targeted Screening**

*Henrik Hupatz^a,b†^, Ida Rahu^a,†^*, Wei-Chieh Wang^a^, Pilleriin Peets^c^, Emma H. Palm^d^, Anneli Kruve^a,b,e^**

^a^ Department of Materials and Environmental Chemistry, Stockholm University, Svante Arrhenius väg 16, 114 18 Stockholm, Sweden

^b^ Stockholm University Center for Circular and Sustainable Systems (SUCCeSS), Stockholm University, 106 91 Stockholm, Sweden

^c^ Institute of Biodiversity, Faculty of Biological Science, Cluster of Excellence Balance of the Microverse, Friedrich Schiller University Jena, 07743, Jena, Germany

^d^ Luxembourg Centre for Systems Biomedicine (LCSB), University of Luxembourg, 6 Avenue du Swing, 4367 Belvaux, Luxembourg

^e^ Department of Environmental Science, Stockholm University, Svante Arrhenius väg 8, 114 18 Stockholm, Sweden

^†^ Authors contributed equally

* Corresponding authors: [ida.rahu@mmk.su.se](mailto:ida.rahu@mmk.su.se) and [anneli.kruve@su.se](mailto:anneli.kruve@su.se)

**Contents**

[Section S1: Chemicals 2](#_Toc172565012)

[Section S2: Liquid Chromatography High-Resolution Mass Spectrometry Analysis 3](#_Toc172565013)

[Section S3: Data Processing 5](#_Toc172565014)

[Section S4: Structural Annotation 8](#_Toc172565015)

[Section S5: Prioritization of Candidate Structures 9](#_Toc172565016)

[Section S6: Chemical Space Visualization 10](#_Toc172565017)

[Section S7: Similarity of Candidate Structures 11](#_Toc172565018)

[Section S8: Visualization of Candidate Annotation and Prioritization 12](#_Toc172565019)

[Section S9: Candidate Structures 15](#_Toc172565020)

[References 35](#_Toc172565021)

# Section S1: Chemicals

All chemicals were purchased in analytical grade. The influent water was provided by the Swedish Environmental Research Institute (Stockholm, Sweden). An overview of all chemicals, including the supplier, can be found in Table S3.

Water (Honeywell Riedel-de Haën™, Seelze, Germany) and acetonitrile (CHROMASOLV™ for HPLC, gradient grade, ≥99.9%) with 0.1% of formic acid (reagent grade, Scharlau, Barcelona, Spain) were used to prepare the mobile phase for LC/IMS/ESI/HRMS measurement.

*CCS* calibration was implemented with mix standard (Major Mix IMS/Tof Calibration Kit | 186008113) obtained from Waters (Waters Corporation, UK).

## Section S2: Liquid Chromatography High-Resolution Mass Spectrometry Analysis

The influent water samples were prepared by filtering through a 0.45 µm filter Filtropur S (Sarstedt AG & Co. KG, Nümbrecht, Germany). Chemicals in Table S3 were dissolved in influent water to obtain the spiked influent sample, and in water to obtain the blank sample. Both samples were analyzed in triplicates with LC/ESI/IM/HRMS system in positive ionization mode. The isomer mixture of *trans*-ferulic acid, dimethyl phthalate, and dimethyl isophthalate was dissolved in water and measured in positive and negative ionization modes. The measurement was conducted on the Waters Select Series Cyclic IMS system with a time-of-flight mass analyzer in V-mode, and the resolution was determined to be 39,460. Chromatographic separation was performed with a Kinetex column (150 x 3 mm, 2.6 µM PS C18 100 Å, Phenomenex, USA). The mobile phase consisted of water and acetonitrile with 0.1% formic acid. The LC gradient started from 5% of acetonitrile and increased to 100% over 20 min. Next, the acetonitrile content was kept at 100% for 5 min and reduced to 5% in 0.1 min. The column was equilibrated at these conditions for 5 min between injections. The mobile phase flow rate was 0.35 mL/min, and the column oven temperature was 40 °C. 10 µL of the sample was injected in each run, and samples were analyzed in triplicates.

For ionization in positive mode, the capillary voltage was 2.30 kV, cone voltage was 20 V, source offset was 10 V, source temperature was 150°C, desolvation temperature was 550 °C, cone gas flow rate was 50 L/h, desolvation gas flow rate was 400 L/h, and nebulizer gas pressure was 6.0 bar. For negative ionization mode, the parameters were kept the same, but a capillary voltage of 2.00 kV was used instead. Leucine enkephalin with a reference *m/z* of 556.27658 in positive mode and *m/z* of 554.26202 in negative mode was infused into the source during the LC analysis and used to apply lockmass correction to all measured *m/z*.

For ion mobility separation with cyclic IM, pushes per bin were 5. The TW static height was 15 V. The gate TW height was 6 V. The TW velocity was 375 m/s. The ions were separated in one cycle, and the setting for the cyclic sequence is shown in Table S1.

**Table S1** Parameters for IM data acquisition sequence

|  | **Time (ms)** | **Pre array** | | **Array** | | | **Post array** | |
| --- | --- | --- | --- | --- | --- | --- | --- | --- |
|  |  | **gradient**  **(V)** | **entrance**  **(V)** | **TW height**  **(V)** | **offset**  **(V)** | **exit**  **(V)** | **gradient**  **(V)** | **bias**  **(V)** |
| Inject | 10 | 85 | 70 | 10 | 2 | 45 | 50 | 35 |
| Separate | 2 | 85 | 70 | 30 | 0 | 70 | 30 | 35 |
| Eject and acquire | 66 | 85 | 70 | 50 | 12 | 45 | 2 | 35 |

The collision cells were operated alternatingly in a low collision energy setting, aiming for as little fragmentation as possible to provide parent ions, and a high collision energy setting to produce fragment ions. The trap collision voltage was kept constant at 5 V in both settings. In the low-energy setting, the transfer collision voltage was 2 V; in the high-energy setting, a ramp from 10 to 55 V was used. The scan time was set to 0.5 s, and the TOF scanned the mass ranging from 50 to 1200 Da. Other optics parameters are recorded in Table S2.

**Table S2** Optics parameters for ESI/IM/HRMS data acquisition

| **Name** | **Value** | **Unit** | **Name** | **Value** | **Unit** |
| --- | --- | --- | --- | --- | --- |
| **RF setting** | | | | | |
| Stepwave RF | 100 | V | Ion guide RF | 400 | V |
| Trap RF | 400 | V | Driftcell RF | 300 | V |
| Pre/post array RF | 350 | V | Cyclic RF | 250 | V |
| Transfer RF | 200 | V |  |  |  |
| **Pre transfer guide** | | | | | |
| Pre trans gradient | 7.0 | V | Pre trans bias | 0.0 | V |
| **Transfer (LINAC)** | | | | | |
| Transfer entrance | 2.0 | V | Transfer gradient | 4.0 | V |
| Transfer exit | 15.0 | -V |  |  |  |
| **Trap** | | | | | |
| Trap TW velocity | 311 | m/s | Trap TW pulse height | 4.0 | V |
| Trap Entrance | 2.0 | V | Trap bias | 2.0 | V |
| Trap DC | -4.0 | V | Trap exit | 0.0 | V |
| **Post trap guide** | | | | | |
| Post trap gradient | 3.0 | V | Post trap bias | 35.0 | V |
| **StepWave** | | | | | |
| Body gradient | 10 | V | Head gradient | 5 | V |
| Ion guide 1 offset | 3.0 | V | Ion guide 2 offset | 0.3 | V |
| IG TW pulse height | 0.2 | V | IG TW Velocity | 150 | m/s |

# Section S3: Data Processing

The raw data were processed with Progenesis QI (V3.0, Waters) and Unifi (V1.9.4.053, Waters). A screening list was applied to label the extracted LC/HRMS features for the spiked chemicals based on the exact mass and retention time previously determined (Table S3). 29 spiked chemicals were automatically labeled by the software across the triplicate injections.

**Table S3** Information about the spiked chemicals and the isomeric mixture, presented as the bottom three entries of the table, highlighted in bold.

| **Chemical name** | **Supplier** | | **Formula** | | **CAS registry**  **number** | | **Conc.**  **(mM)** | | **RT**  **(min)** | |
| --- | --- | --- | --- | --- | --- | --- | --- | --- | --- | --- |
| 3-amino-1,2,4-triazol (amitrole) | Sigma-Aldrich, Germany | | C2H4N4 | | 61-82-5 | | 6.29 | | ND* | |
| aspartame | Sigma-Aldrich, Germany | | C14H18N2O5 | | 22839-47-0 | | 1.73 | | 5.02 | |
| atrazine | Sigma-Aldrich, Germany | | C8H14ClN5 | | 1912-24-9 | | 2.76 | | 10.73 | |
| abamectin (avermectin B1a) | Sigma-Aldrich, Germany | | C48H72O14 | | 71751-41-2 | | 0.72 | | 18.52 | |
| 1H-benzotriazole | Sigma-Aldrich, Germany | | C6H5N3 | | 95-14-7 | | 4.82 | | ND* | |
| butocarboxim | Sigma-Aldrich, Germany | | C7H14N2O2S | | 34681-10-2 | | 1.69 | | 8.36 | |
| caffeine | Merck, USA | | C8H10N4O2 | | 58-08-2 | | 3.01 | | 5.16 | |
| carbamazepine | Sigma-Aldrich, Germany | | C15H12N2O | | 298-46-4 | | 2.29 | | 9.70 | |
| cefoperazone sodium salt | Sigma-Aldrich, Germany | | C25H26N9NaO8S2 | | 62893-20-3 | | 0.96 | | 7.78 | |
| chlormequat chloride | Sigma-Aldrich, Germany | | C5H13ClN+ | | 999-81-5 | | 4.27 | | 1.70 | |
| chlorothiazide | Sigma-Aldrich, Germany | | C7H6ClN3O4S2 | | 58-94-6 | | 1.94 | | 4.90 | |
| clarithromycin | Sigma-Aldrich, Germany | | C38H69NO13 | | 81103-11-9 | | 0.66 | | 8.73 | |
| dichlorvos | Sigma-Aldrich, Germany | | C4H7Cl2O4P | | 62-73-7 | | 2.35 | | 9.47 | |
| dimethyl phthalate | Aldrich, Germany | | C10H10O4 | | 131-11-3 | | 2.56 | | 9.89 | |
| diphenyl phthalate | Sigma-Aldrich, Germany | | C20H14O4 | | 84-62-8 | | 1.57 | | 15.71 | |
| efavirenz | Sigma-Aldrich, Germany | | C14H9ClF3NO2 | | 154598-52-4 | | 1.78 | | 14.14 | |
| emamectin benzoate (B1a) | Sigma-Aldrich, Germany | | C49H75NO13 | | 155569-91-8 | | 0.55 | | 11.93 | |
| *N*-guanylurea sulfate salt hydrate | Aldrich, Germany | | C4H16O7N8S | | 207300-86-5 | | 1.50 | | ND* | |
| haloperidol | Sigma-Aldrich, Germany | | C21H23ClFNO2 | | 52-86-8 | | 1.54 | | 7.72 | |
| histamine | Sigma-Aldrich, Germany | | C5H9N3 | | 51-45-6 | | 5.65 | | ND* | |
| imazalil | Fluka, Germany | | C14H14Cl2N2O | | 35554-44-0 | | 1.69 | | 8.03 | |
| ivermectin (B1a) | Sigma-Aldrich, Germany | | C48H74O14 | | 70288-86-7 | | 0.58 | | ND* | |
| L-alanine | Sigma-Aldrich, Germany | | C3H7NO2 | | 56-41-7 | | 5.49 | | ND* | |
| L-phenylalanine | Sigma-Aldrich, Germany | | C9H11NO2 | | 63-91-2 | | 3.22 | | ND* | |
| methamidophos | Sigma-Aldrich, Germany | | C2H8NO2PS | | 10265-92-6 | | 3.57 | | ND* | |
| metolachlor | Aldrich, Germany | | C15H22ClNO2 | | 51218-45-2 | | 2.05 | | 14.03 | |
| nigericin sodium salt | Sigma-Aldrich, Germany | | C40H67NaO11 | | 28643-80-3 | | 16.33 | | ND* | |
| **Chemical name** | | **Supplier** | | **Formula** | | **CAS registry**  **number** | | **Conc.**  **(mM)** | | **RT**  **(min)** |
| octocrylene | | Aldrich, Germany | | C24H27NO2 | | 6197-30-4 | | 1.64 | | 19.01 |
| progesterone | | Sigma-Aldrich, Germany | | C21H30O2 | | 57-83-0 | | 1.53 | | 15.02 |
| rifaximin | | Aldrich, Germany | | C43H51N3O11 | | 80621-81-4 | | 0.63 | | 13.31 |
| saccharin | | Sigma-Aldrich, Germany | | C7H5NO3S | | 81-07-2 | | 2.97 | | ND* |
| simazine | | Riedel-de-Haen, Germany | | C7H12ClN5 | | 122-34-9 | | 2.52 | | 9.24 |
| spinosad (A) | | Sigma-Aldrich, Germany | | C41H65NO10 | | 168316-95-8 | | 0.84 | | 10.89 |
| sucralose | | Sigma-Aldrich, Germany | | C12H19Cl3O8 | | 56038-13-2 | | 1.26 | | 5.63 |
| 2-(thiocyanatomethylthio)benzothiazole  (TCMTB) | | Honeywell Fluka, Germany | | C9H6N2S3 | | 21564-17-0 | | 1.98 | | 13.18 |
| tetraethylammonium iodide | | Sigma-Aldrich, Germany | | C8H20N+ | | 68-05-3 | | 2.02 | | ND* |
| tetrahexylammonium benzoate | | Lodak, USA | | C24H52N+ | | 16436-29-6 | | 1.57 | | 13.69 |
| trichlorfon | | Aldrich, Germany | | C4H8Cl3O4P | | 52-68-6 | | 2.27 | | 6.46 |
| tylosin phosphate | | Sigma-Aldrich, Germany | | C46H80NO21P | | 1405-53-4 | | 0.60 | | 8.16 |
| uracil | | Merck, USA | | C4H4N2O2 | | 66-22-8 | | 4.64 | | ND* |
| vancomycin hydrochloride | | Sigma-Aldrich, Germany | | C66H75Cl2N9O24 | | 1404-90-6 | | 0.33 | | 3.51 |
| **dimethyl isophthalate** | | **Sigma-Aldrich, Germany** | | **C10H10O4** | | **1459-93-4** | | **2.43** | | **11.25** |
| **dimethyl phthalate** | | **Aldrich, Germany** | | **C10H10O4** | | **131-11-3** | | **2.43** | | **9.95** |
| ***trans*-ferulic acid** | | **Sigma-Aldrich, Germany** | | **C10H10O4** | | **537-98-4** | | **2.43** | | **6.95** |

* ND, not detected

To ensure accurate fragmentation tree computation and subsequent formula annotation with SIRIUS+CSI:FingerID [1, 2], the following criteria were employed to identify unknown peaks: a) absence in the blank sample, b) higher average intensity across triplicate injections, and c) presence of informative MS^2^ spectra. Peaks meeting these criteria were manually evaluated and selected as "truly unknowns" for further analysis. MS^1^ and MS^2^ spectra for both spiked standards and selected unknown LC/HRMS features were aligned based on the drift time between parent and product ions, and the obtained spectra were saved as Microsoft Excel files with Unifi.

To extract pertinent information from spectral files, an R workflow was developed. Initially, the workflow conducts a detailed spectral matching process, aligning each LC/HRMS feature with its corresponding MS^1^ and MS^2^ spectra. MS^1^ spectra are aligned based on the observed *m*/*z* values provided in a comprehensive feature table within the original file. Alignment requires the peak with the highest intensity in the MS^1^ spectrum to match the observed *m*/*z* within a mass error range of ±0.0005. Any MS^1^ spectra failing to align with the specified mass in the feature table are flagged for manual inspection, typically indicative of in-source fragmentation or unusual adduct formation phenomena. Furthermore, LC/HRMS features lacking MS^2^ spectra are also flagged for further attention.

Subsequently, data obtained from triplicate measurements are integrated using hierarchical clustering, employing an agglomerative approach with Euclidean distance via the *hclust()* function [3]. Clustering is performed based on observed *m*/*z*, retention time (RT), and collision cross section (*CCS*) values. Finally, all the relevant data regarding each LC/HRMS feature, including raw spectra and mean *m*/*z*, RT, and *CCS* values derived from triplicate measurements, are gathered and stored in designated folders.

To illustrate the methods employed in non-targeted screening (NTS) for structure annotation and prioritization of candidate structures, we selected 10 LC/HRMS features representing spiked chemicals and 10 truly unknown features from the obtained spectral dataset. Initially, we excluded features lacking MS^2^ spectra from consideration. Subsequently, to facilitate RT prediction using the RTI model [4, 5], we set aside spiked chemicals required for building the calibration curve of the model, as specified by the model developers. Additionally, LC/HRMS features with parent ion mass exceeding 850 Da were discarded to optimize the efficiency of SIRIUS+CSI:FingerID calculations. From the remaining LC/HRMS features, we selected the spiked chemicals and truly unknown features through a random split using the *train_test_split()* function [6] from the Python module *scikit-learn* [7]. During this process, it was crucial to ensure that our illustrative dataset encompassed a broad spectrum of *m*/*z*, RT, and *CCS* values while maintaining randomness and versatility. To achieve this, we standardized these parameters using the *MinMaxScaler()* function [8] from *scikit-learn* before the split. This approach aimed to establish uniformity and comparability across features with varying numerical ranges, a pivotal step for mitigating biases and ensuring that the dataset accurately reflected the overall distribution of these parameters. All the information about selected LC/HRMS features is given in Table S4.

**Table S4** Information about selected LC/HRMS features

| **LC/HRMS feature** | **Type** | ***m*/*z*** | **Experimental RT**  **(min)** | **Experimental *CCS***  **(Å^2^)** |
| --- | --- | --- | --- | --- |
| Feature_163.0381 | spiked | 163.0381 | 9.90 | 123.51 |
| Feature_195.0875 | spiked | 195.0875 | 5.16 | 137.56 |
| Feature_198.9398 | truly unknown | 198.9398 | 1.46 | 141.55 |
| Feature_202.0855 | spiked | 202.0855 | 9.26 | 142.88 |
| Feature_216.1012 | spiked | 216.1012 | 10.76 | 149.00 |
| Feature_237.1026 | spiked | 237.1026 | 9.70 | 149.28 |
| Feature_284.1417 | spiked | 284.1417 | 14.07 | 159.61 |
| Feature_297.0569 | spiked | 297.0569 | 8.08 | 165.46 |
| Feature_315.2322 | spiked | 315.2322 | 15.04 | 178.79 |
| Feature_327.1565 | truly unknown | 327.1565 | 15.30 | 171.33 |
| Feature_376.1487 | spiked | 376.1487 | 7.75 | 192.31 |
| Feature_388.2547 | truly unknown | 388.2547 | 5.24 | 183.44 |
| Feature_401.2872 | truly unknown | 401.2872 | 15.46 | 195.42 |
| Feature_411.1984 | truly unknown | 411.1984 | 13.61 | 189.14 |
| Feature_432.2812 | truly unknown | 432.2812 | 5.54 | 191.64 |
| Feature_595.3495 | truly unknown | 595.3495 | 7.54 | 233.30 |
| Feature_597.3648 | truly unknown | 597.3648 | 12.25 | 226.87 |
| Feature_644.4951 | truly unknown | 644.4951 | 17.37 | 256.46 |
| Feature_688.5216 | truly unknown | 688.5216 | 17.32 | 265.35 |
| Feature_748.4870 | spiked | 748.4870 | 8.77 | 265.81 |

## Section S4: Structural Annotation

As an example of spectral library matching, MassBank Europe [9] was quarried based on the averaged peak list. For this purpose, the MS^2^ spectra from different acquisitions were averaged within a mass accuracy of 0.01 Da. The relative intensity threshold was set to 5%, and all MS^2^ spectra in positive ESI mode were queried independently of the adduct type. For each LC/HRMS feature, the top ten spectra were selected based on their cosine similarity, regardless of the absolute cosine similarity values. This selection may include multiple spectra for a single chemical within one LC/HRMS feature.

*In silico* structural annotation was performed with MetFrag [10, 11] through the web interface (version 2.5.0). The candidate structures were searched from PubChem based on the exact mass of the parent ion with 10 ppm mass accuracy. The considered adduct types included [M]^+^, [M+H]^+^, [M+NH_4_]^+^, [M+Na]^+^, and [M+K]^+^. For MS^2^ matching, the tree depth of 2 was considered. The ten candidate structures were selected based on the MetFrag score across all considered adduct types.

We employed SIRIUS+CSI:FingerID (version 5.8.6) for structural library matching, leveraging information extracted from MS^2^ spectra. To facilitate this process, we created ".ms" files for each LC/HRMS feature following the SIRIUS+CSI:FingerID documentation guidelines [12]. These files contained raw MS^1^ and MS^2^ peak lists extracted from all triplicates. Where possible, we also included the peak list of merged MS^1^ spectra. To generate these merged spectra, we utilized the *averageSpectraSingle()* function from the R library *msPurity* [13, 14], employing hierarchical clustering with mean averaging. Additionally, in line with the NTS approach, we specified the ionization mode [M+?]^+^ to encompass various ionization types. The generated ".ms" files were utilized as inputs for SIRIUS to perform structural annotation. Although default values were predominantly employed for most parameters, we chose not to restrict detectable elements and focused solely on the adduct types [M]^+^, [M+H]^+^, [M+NH_4_]^+^, [M+Na]^+^, and [M+K]^+^ (similarly to MetFrag approach) during the analysis.

For *in silico* candidate structure generation, Spec2Mol [15] was utilized, sourced from GitHub [16], and implemented according to the guidelines provided. This model takes mass spectra as input and generates a “.csv” file containing the candidate structures represented as SMILES notations. In this study, 10 structures were generated for each LC/HRMS feature. To prepare the input spectra for each LC/HRMS feature, MS^1^ and MS^2^ spectra from three acquisitions were averaged within a mass accuracy of 0.01 Da. In this framework, the averaged MS^1^ spectra served as positive mode low-energy spectra input, while the averaged MS^2^ spectra served as positive mode high-energy spectra input. Due to the fact that Spec2Mol was not trained for structures with m/z > 500 Da, no candidate structures were obtained for LC/HRMS features exceeding this value.

The SMILES notations of all obtained candidate structures were standardized, and any invalid or duplicated structures within each LC/HRMS feature and method pair were eliminated. Not all four methods yielded 10 candidate structures for each LC/HRMS feature. The candidate structures for all methods and LC/HRMS features were combined, resulting in 515 unique candidate structures. Seven candidate structures were suggested by two different methods for the same LC/HRMS feature. These candidate structures are represented by dual-colored circles in Figure 7A and in Figures S1A–S3A. A table containing all candidate structures and their predicted RT and *CCS* values is available in the Table Supplementary Information 2 and on GitHub (<https://github.com/kruvelab/NTS_LC_HRMS>). The structures of all candidates are depicted in SI1 Section S9.

## Section S5: Prioritization of Candidate Structures

Candidate structures were prioritized utilizing predicted RT and *CCS* values. To predict RT values, we employed the RTI model through the Retention Time Indices Platform [4, 5]. This model necessitates the construction of a calibration curve that maps the experimental RT values to experimental RTI values, utilizing defined calibrants, the details of which are provided in Table S5. The calibration curve was established using the auto-calibrate mode, resulting in the equation RTI = 53.5008 · TR − 62.9247 with an R^2^ value of 0.949. Subsequently, to predict RTs for all candidate structures, relevant files were generated based on RTI documentation, and calculations were performed using batch mode with uncertainty measurement “OTrAMS” [17]. Candidate structures were then prioritized based on a cutoff criterion of ±2 standard residuals, corresponding to the applicability domain defined by “box1” and “box2” in the output files.

**Table S5** Calibrants and their experimental RT (min) used for building the calibration curve for the RTI model.

| **Calibrant** | **Experimental RT (min)** |
| --- | --- |
| chlormequat | 1.87 |
| vancomycin | 3.54 |
| cefoperazone | 7.76 |
| trichlorfon | 6.47 |
| butocarboxim | 8.37 |
| dichlorvos | 9.48 |
| tylosin | 8.19 |
| TCMTB | 13.20 |
| rifaximin | 13.33 |
| spinosad_A | 10.92 |
| emamectin | 11.97 |
| avermectin | 18.55 |

*CCS* values for candidate structures were determined using CCSbase [18, 19], taking into consideration the specific adduct type (version 1.2). Prioritization was based on achieving a 3% agreement between the experimentally measured *CCS* values for unknown LC/HRMS features and the predicted *CCS* values for candidate structures.

## Section S6: Chemical Space Visualization

To visualize the coverage of chemical space across commonly used datasets for annotating LC/HRMS features or prioritizing candidate structures in NTS workflows, we opted to use the PubChemLite (version 0.3.0) dataset [20] as a representative sample of the chemical diversity encountered during analysis. To ensure compatibility with LC/ESI/HRMS analysis, we preprocessed the PubChemLite dataset by filtering out chemicals with a monoisotopic mass below 50 Da and those lacking any carbon atoms.

In this study, we focused on two datasets used for LC/HRMS feature annotation: ESI+ HRMS data from MassBank Europe (version 2023.11) (referred to as MassBank) [9], widely employed for spectral library matching, and the positive mode training data of SIRIUS+CSI:FingerID, utilized for structural library matching within the field. Additionally, we considered two datasets relevant for prioritizing candidate structures: the training data of the positive mode RTI model and the training data of CCSbase [21].

To ensure alignment with the NTS LC/ESI/HRMS approach, all these datasets, including PubChemLite, underwent the same Python data processing pipeline, leveraging functions from the open-source cheminformatics software RDKit [22]. Initially, the isotope labeling of the atoms was removed by adjusting the atom isotope labeling parameter of the function *SetIsotope()* from the *rdkit.Chem.rdchem* [23] module to “0”. Next, disconnected structures within the dataset were identified, and all inorganic ions (e.g., Na^+^, K^+^, Ca^2+^, NO_3_^−^, SO_4_^2−^, Cl^−^, Br^−^) and solvent molecules (e.g., H_2_O, ammonia) were excluded. The remaining ions were neutralized based on the valency of the atoms, accomplished through the utilization of the *rdkit.Chem.SaltRemover* module [24] and *neutralize_atoms()* function [25], alongside the *Uncharger()* function from the *rdkit.Chem.MolStandardize.rdMolStandardize* module [26]. Finally, information about stereochemistry was discarded using the *RemoveStereochemistry()* function from the *rdkit.Chem.rdmolops* module [27]. Moreover, expert knowledge was applied to individually assess the suitability of chemicals containing atoms with negative charges for positive mode ESI analysis.

After this pipeline, all duplicates were removed, resulting in 370,167 unique chemicals represented in SMILES notation for the PubChemLite dataset, 4,310 unique chemicals for the MassBank dataset, 21,188 unique chemicals for the SIRIUS+CSI:FingerID dataset, 1,426 unique chemicals for the RTI dataset, and 4,771 unique chemicals for the CCSbase dataset. To calculate the Uniform Manifold Approximation and Projection (UMAP) [28] embeddings for visualizing the chemical space, binary molecular fingerprints were computed for all these chemicals based on their SMILES notation, utilizing the complete set of SMARTS corresponding to the molecular fingerprint features computed by SIRIUS+CSI:FingerID and leveraging the R package rcdk [29]. This fingerprint type was chosen because its features directly correspond to the structural patterns extractable from HRMS data through SIRIUS+CSI:FingerID. From all the computed features, only the 3878 fingerprint features consistent for positive ESI mode in SIRIUS+CSI:FingerID were retained, referred to as positive mode SIRIUS fingerprint.

The UMAP embedding was learned using the positive mode SIRIUS fingerprint of all the chemicals in the PubChemLite dataset through the Python package umap-learn [30], with parameters set as follows: *n_neighbors* = 50, *min_dist* = 0.5, *random_state* = 42. Afterward, the resulting UMAP embedding was applied to all the other datasets, as depicted in Figure 3. To visualize the candidate structures in the chemical space (refer to Figure 7B and Figures S1B–S3B), the positive mode SIRIUS fingerprint was computed for all of them based on their SMILES representation, followed by the application of the previously learned UMAP embedding.

## Section S7: Similarity of Candidate Structures

To compare the results of various approaches employed for annotating the LC/HRMS features (including experimental and *in silico* spectra matching with MassBank and MetFrag, as well as utilizing SIRIUS+CSI:FingerID and Spec2Mol), a structural similarity analysis was conducted. This involved calculating pairwise Tanimoto similarities between all candidate structures (a total of 522 candidate structures for 20 LC/HRMS features, with 7 structures overlapping between different LC/HRMS features) based on their positive mode SIRIUS fingerprint (Section S6). The *TanimotoSimilarity()* function from the RDKit module *rdkit.DataStructs.cDataStructs* [31] in Python was employed for this calculation. Subsequently, the averaged similarity score was calculated based on all pairwise Tanimoto similarities within each experimental feature, and the results were visualized as a heatmap, with each data point representing these values. Furthermore, overall similarity scores were computed to represent the similarities among all candidates proposed by the methods employed. These scores encompassed the similarity of all candidate structures proposed within a method, as well as the pairwise similarity between structures suggested by different methods.

## Section S8: Visualization of Candidate Annotation and Prioritization


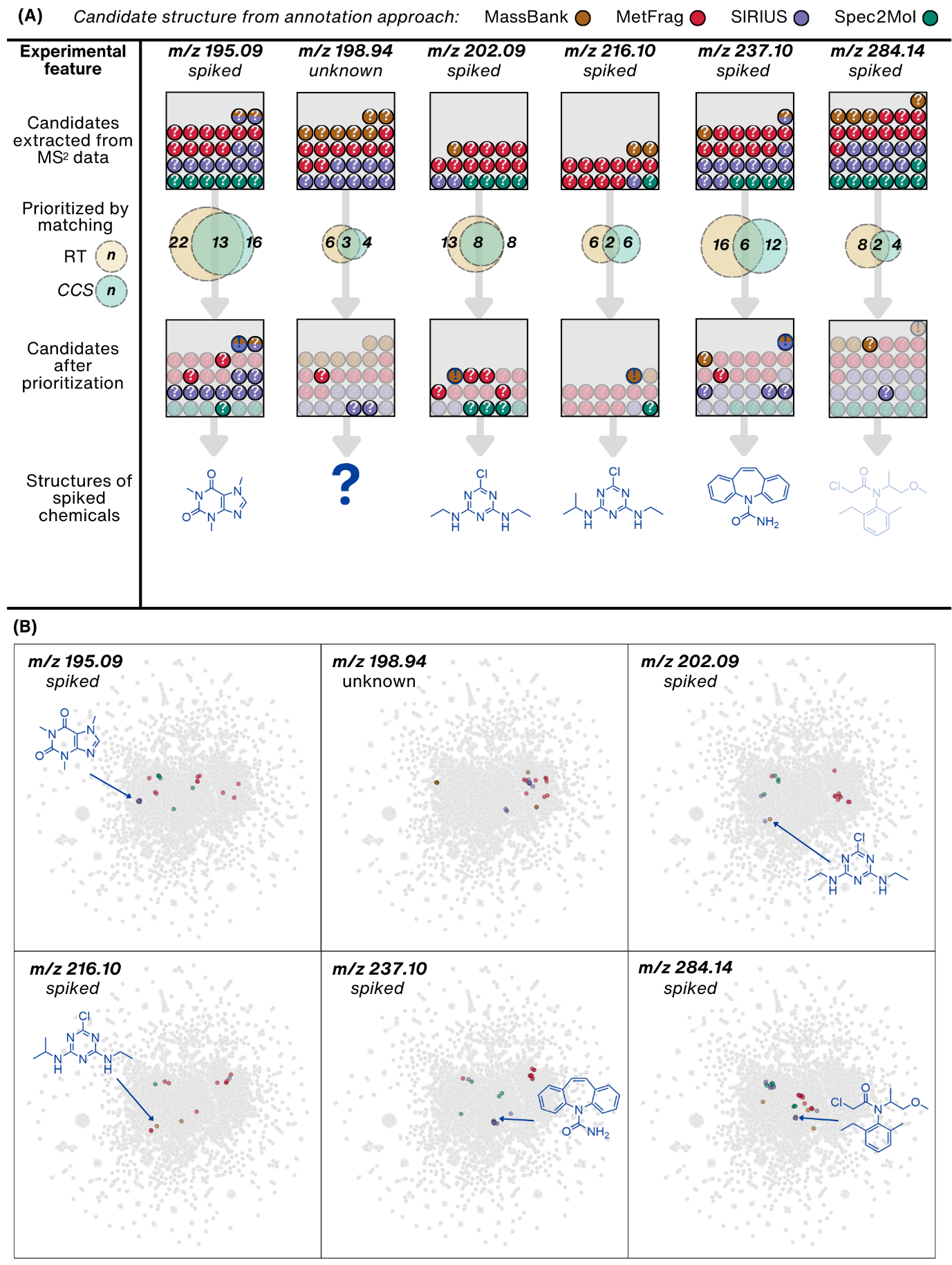


**Figure S1** Visualization of the structural annotation and candidate structure prioritization results for six LC/HRMS features out of the 20 LC/HRMS features studied. (A) The number of candidate structures obtained from experimental and *in silico* spectra matching with MassBank and MetFrag, and by employing SIRIUS+CSI:FingerID and Spec2Mol. Each candidate structure is represented by a colored circle, with the order indicating its rank within the annotation approach. Dual-colored circles represent candidate structures suggested by two methods. The middle panel illustrates the number of candidate structures prioritized based on predicted RT obtained by utilizing the RTI model and *CCS* obtained by employing the CCSbase model. For features corresponding to the spiked chemicals, the correct structure is highlighted with a dark blue exclamation mark. For spiked feature *m/z* 284.14, the correct candidate structure was deprioritized due to mismatching *CCS* values. (B) Visualization of the candidate structures in the chemical space using the UMAP embedding of PubChemLite (Figure 3). All points are transparent, resulting in a darker color when data points are overlaid.


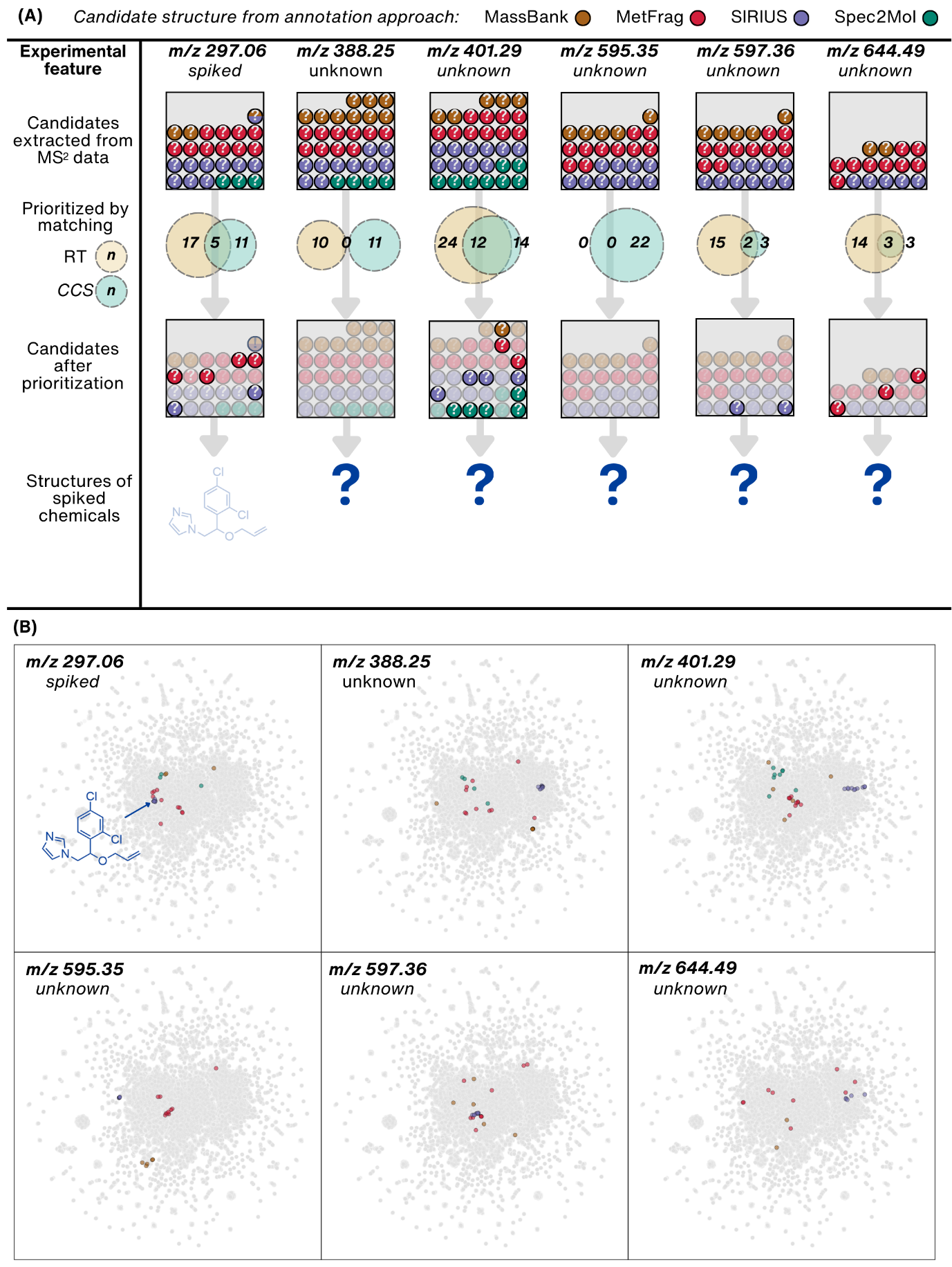


**Figure S2** Visualization of the structural annotation and candidate structure prioritization results for six LC/HRMS features out of the 20 LC/HRMS features studied. (A) The number of candidate structures obtained from experimental and *in silico* spectra matching with MassBank and MetFrag, and by employing SIRIUS+CSI:FingerID and Spec2Mol. Each candidate structure is represented by a colored circle, with the order indicating its rank within the annotation approach. Dual-colored circles represent candidate structures suggested by two methods. The middle panel illustrates the number of candidate structures prioritized based on predicted RT obtained by utilizing the RTI model and *CCS* obtained by employing the CCSbase model. For features corresponding to the spiked chemicals, the correct structure is highlighted with a dark blue exclamation mark. For spiked feature *m/z* 297.06, the correct candidate structure was deprioritized due to mismatching RT. (B) Visualization of the candidate structures in the chemical space using the UMAP embedding of PubChemLite (Figure 3). All points are transparent, resulting in a darker color when data points are overlaid.


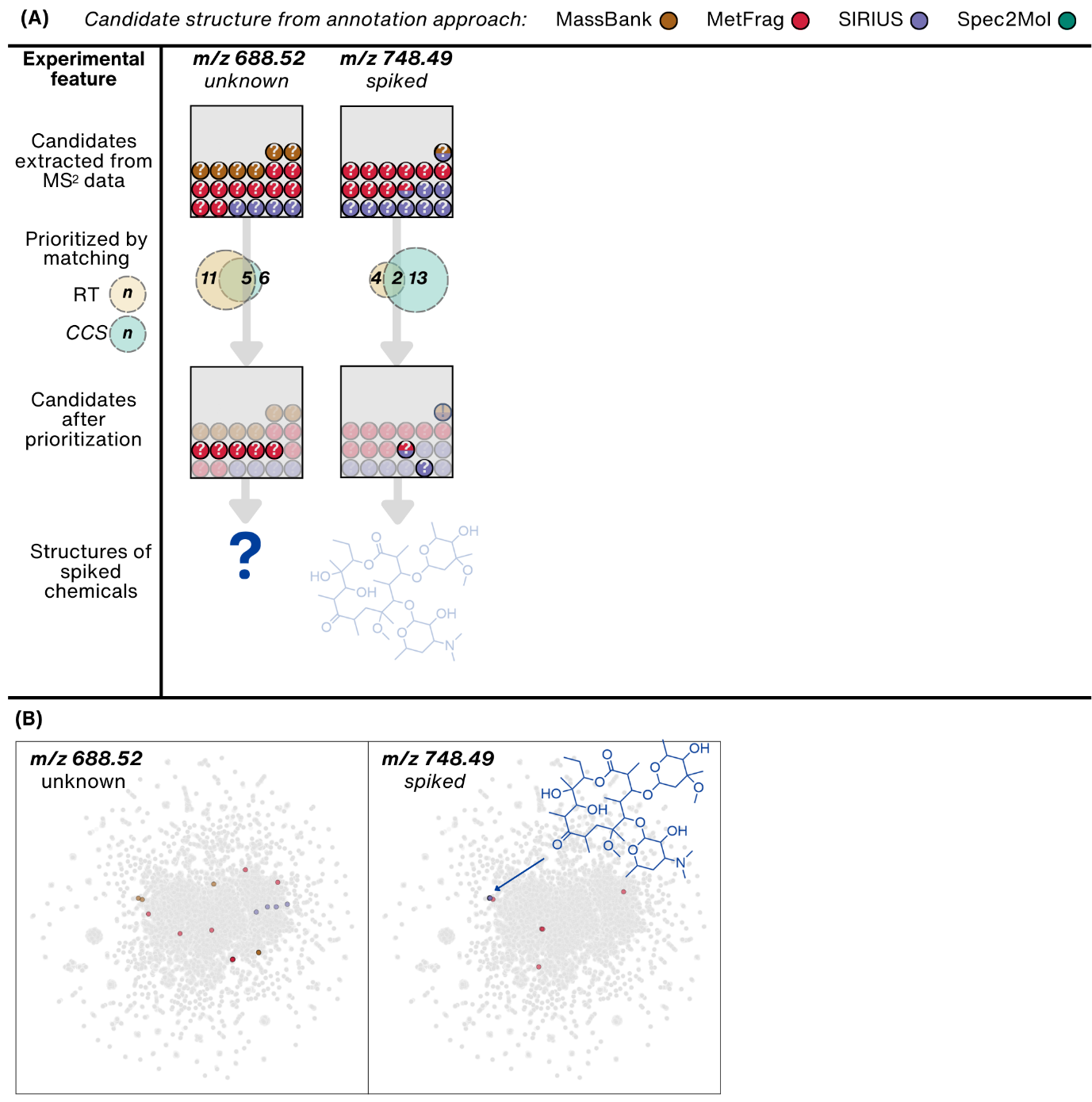


**Figure S3** Visualization of the structural annotation and candidate structure prioritization results for two LC/HRMS features out of the 20 LC/HRMS features studied. (A) The number of candidate structures obtained from experimental and *in silico* spectra matching with MassBank and MetFrag, and by employing SIRIUS+CSI:FingerID and Spec2Mol. Each candidate structure is represented by a colored circle, with the order indicating its rank within the annotation approach. Dual-colored circles represent candidate structures suggested by two methods. The middle panel illustrates the number of candidate structures prioritized based on predicted RT obtained by utilizing the RTI model and *CCS* obtained by employing the CCSbase model. For features corresponding to the spiked chemicals, the correct structure is highlighted with a dark blue exclamation mark. For spiked feature *m/z* 748.49, the correct candidate structure was deprioritized due to mismatching RT. (B) Visualization of the candidate structures in the chemical space using the UMAP embedding of PubChemLite (Figure 3). All points are transparent, resulting in a darker color when data points are overlaid.

## Section S9: Candidate Structures


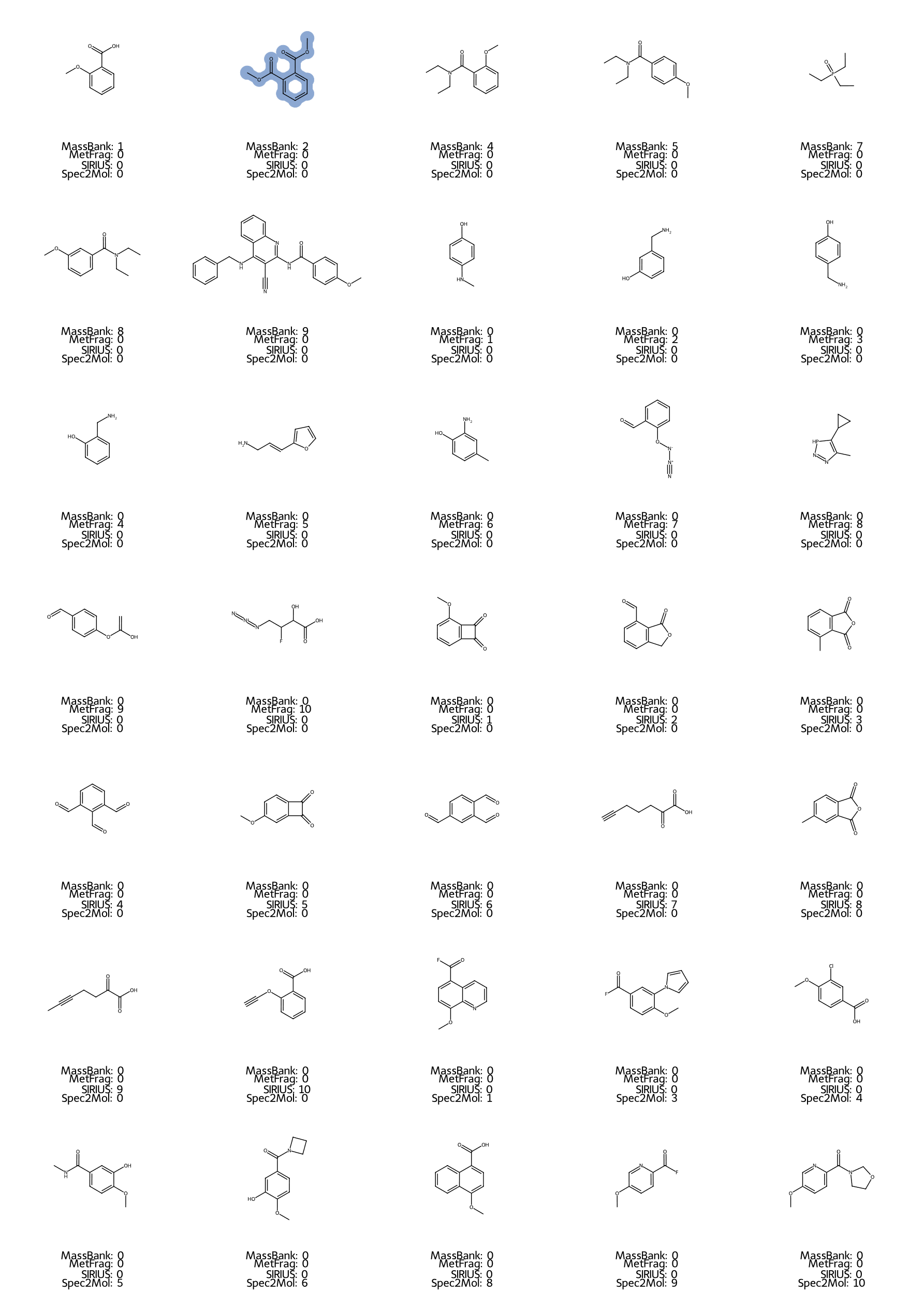


**Figure S4** Candidate structures for spiked feature with *m/z* 163.0381 and rank in annotation method. The correct structure is highlighted in blue.


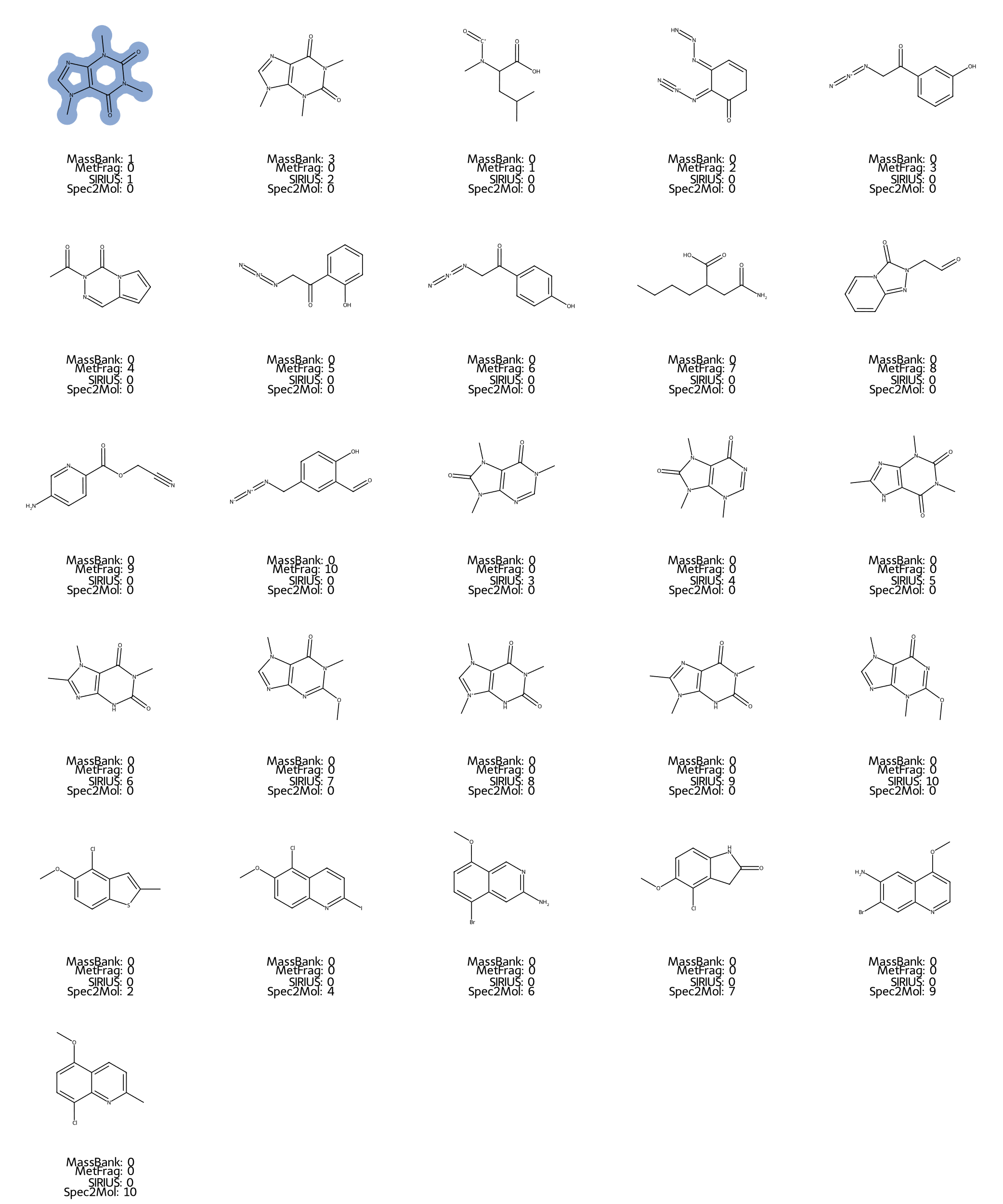


**Figure S5** Candidate structures for spiked feature with *m/z* 195.0875 and rank in annotation method. The correct structure is highlighted in blue.


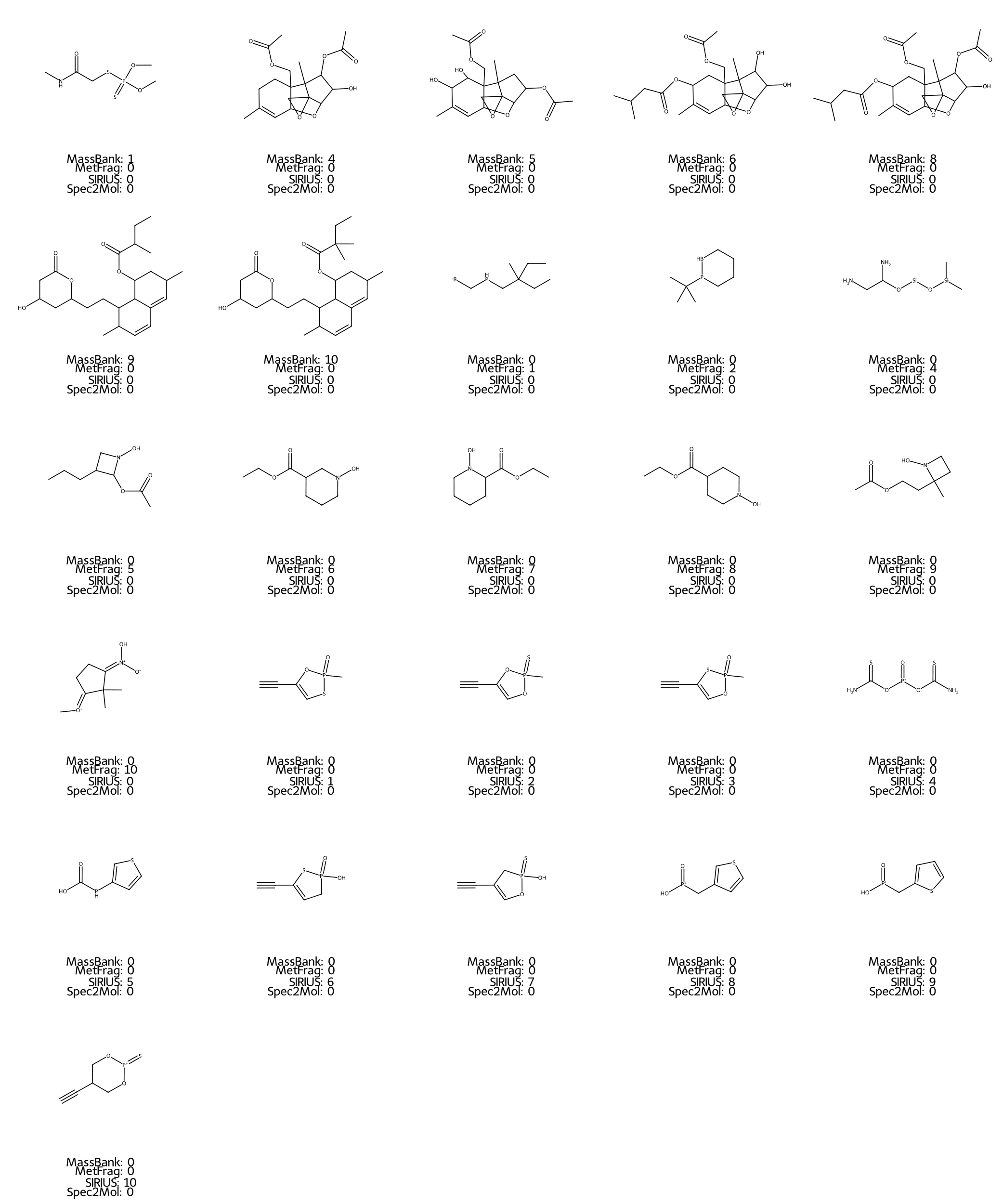


**Figure S6** Candidate structures for unknown feature with *m/z* 198.9398 and rank in annotation method.


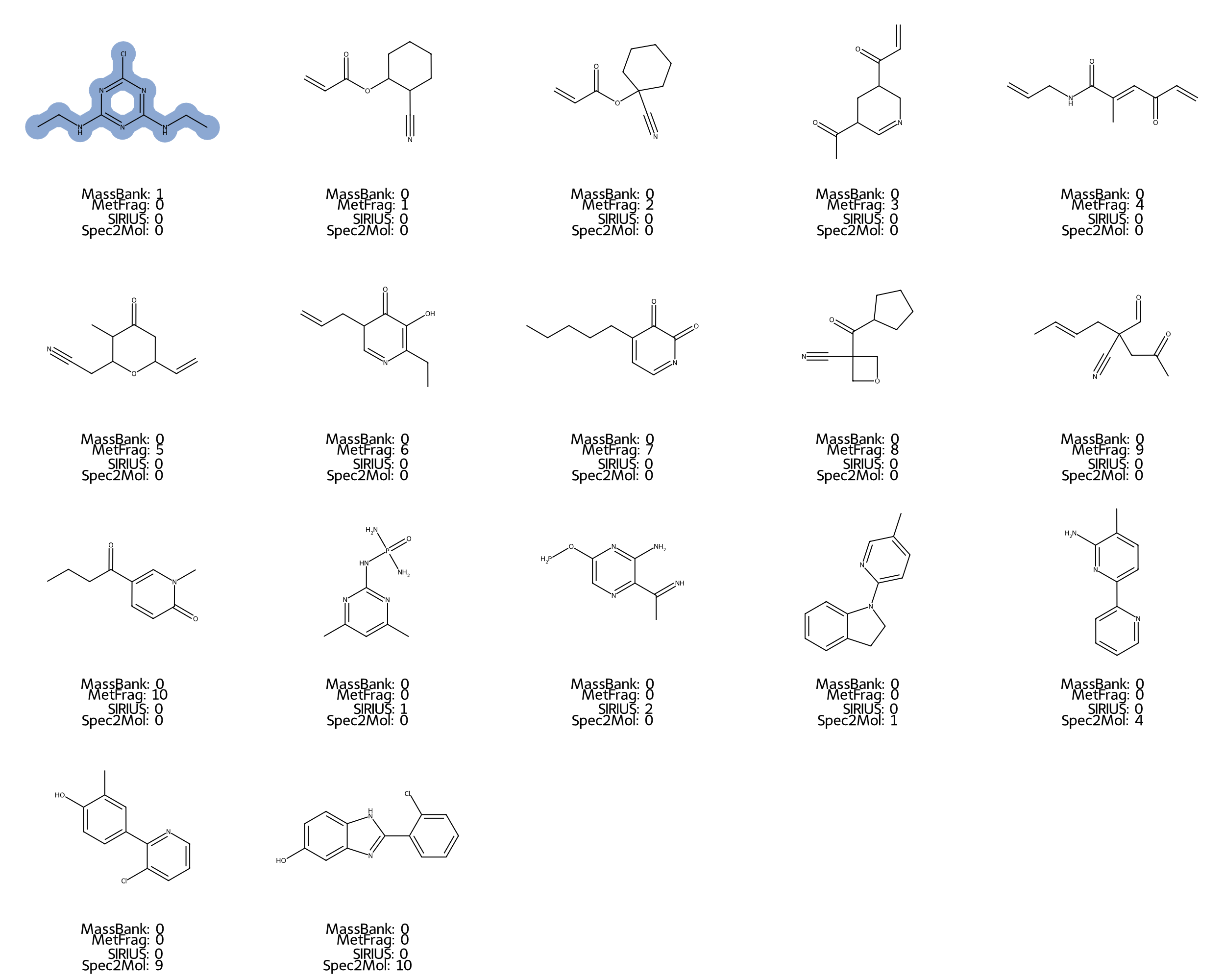


**Figure S7** Candidate structures for spiked feature with *m/z* 202.0855 and rank in annotation method. The correct structure is highlighted in blue.


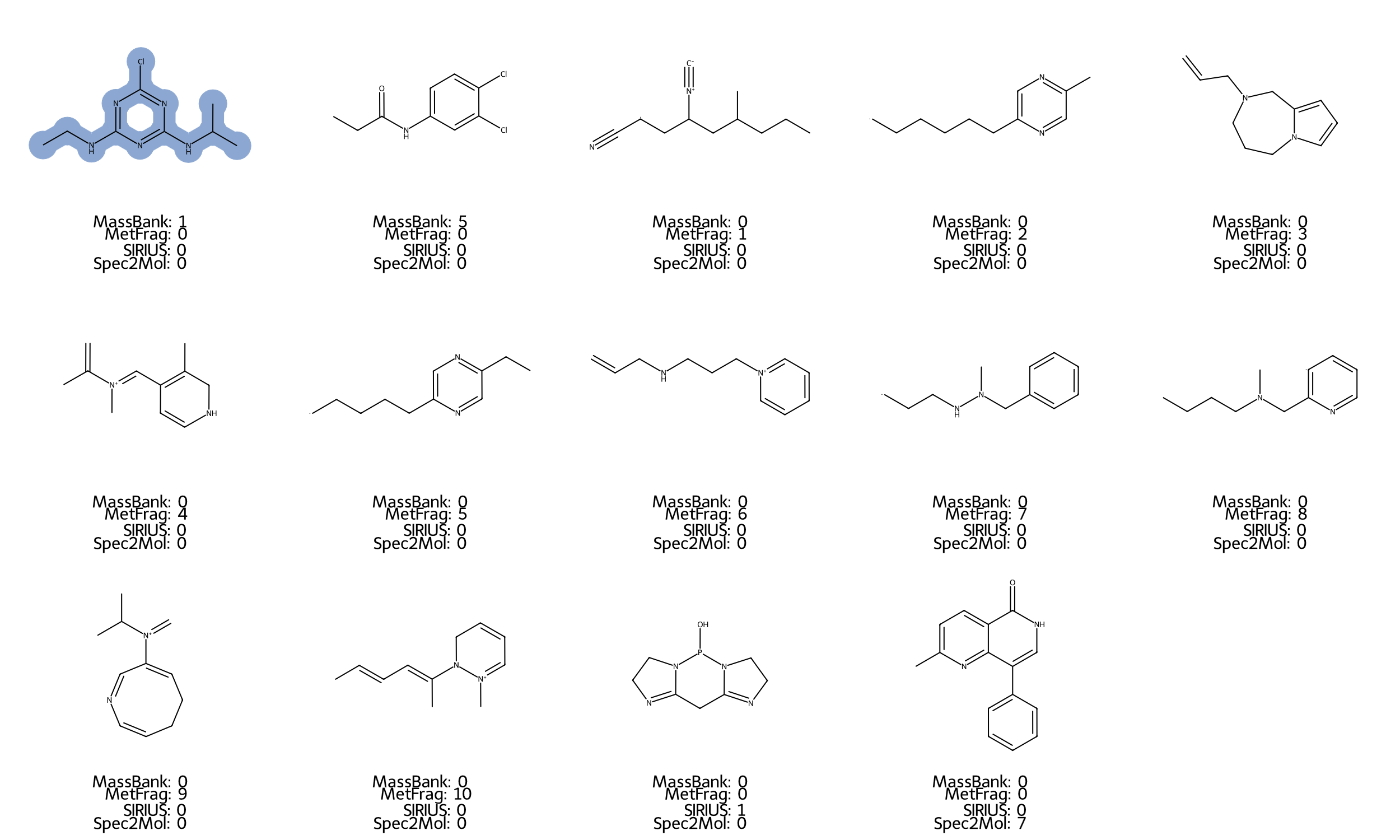


**Figure S8** Candidate structures for spiked feature with *m/z* 216.1012 and rank in annotation method. The correct structure is highlighted in blue.


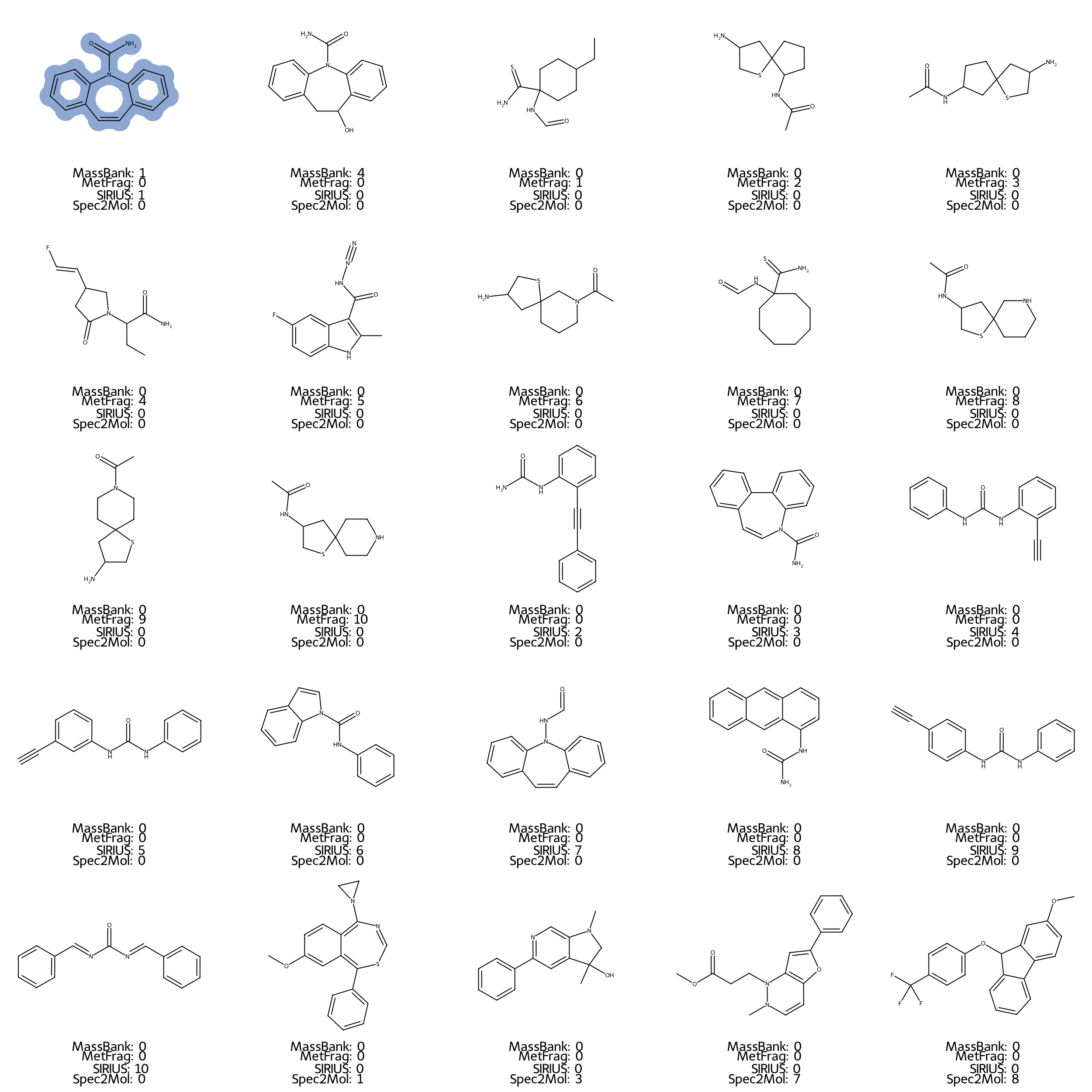


**Figure S9** Candidate structures for spiked feature with *m/z* 237.1026 and rank in annotation method. The correct structure is highlighted in blue.


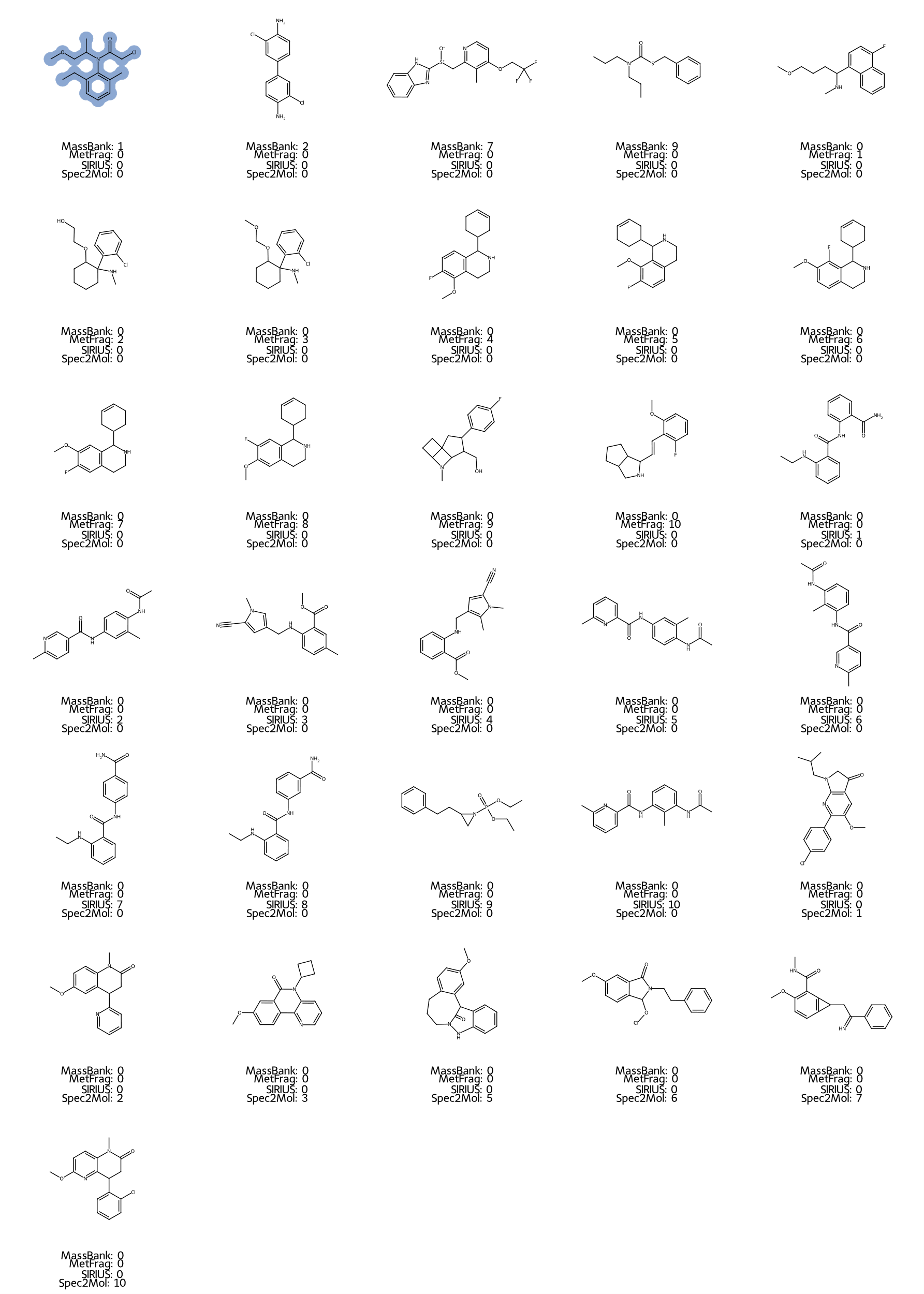


**Figure S10** Candidate structures for spiked feature with *m/z* 284.1417 and rank in annotation method. The correct structure is highlighted in blue.


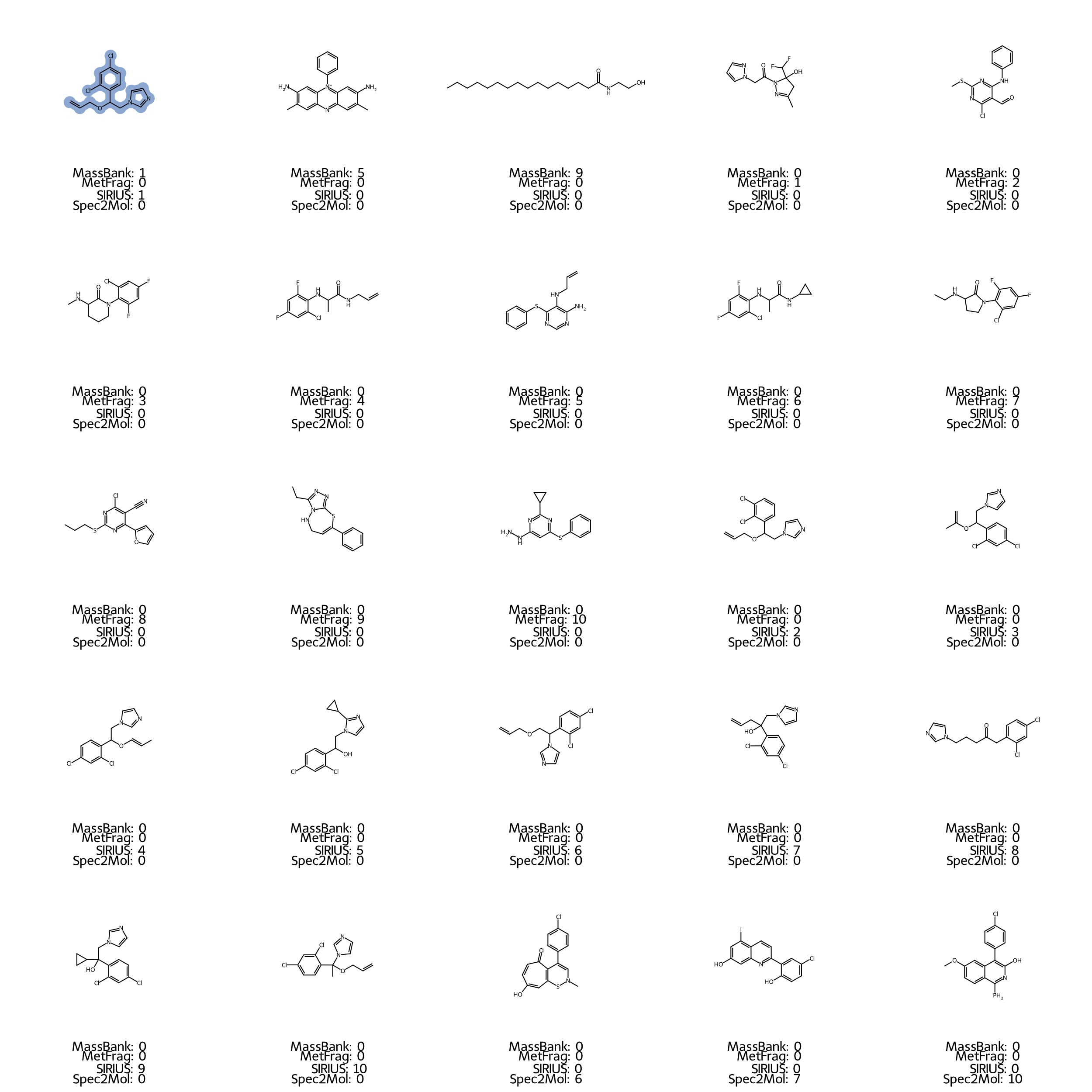


**Figure S11** Candidate structures for spiked feature with *m/z* 297.0569 and rank in annotation method. The correct structure is highlighted in blue.


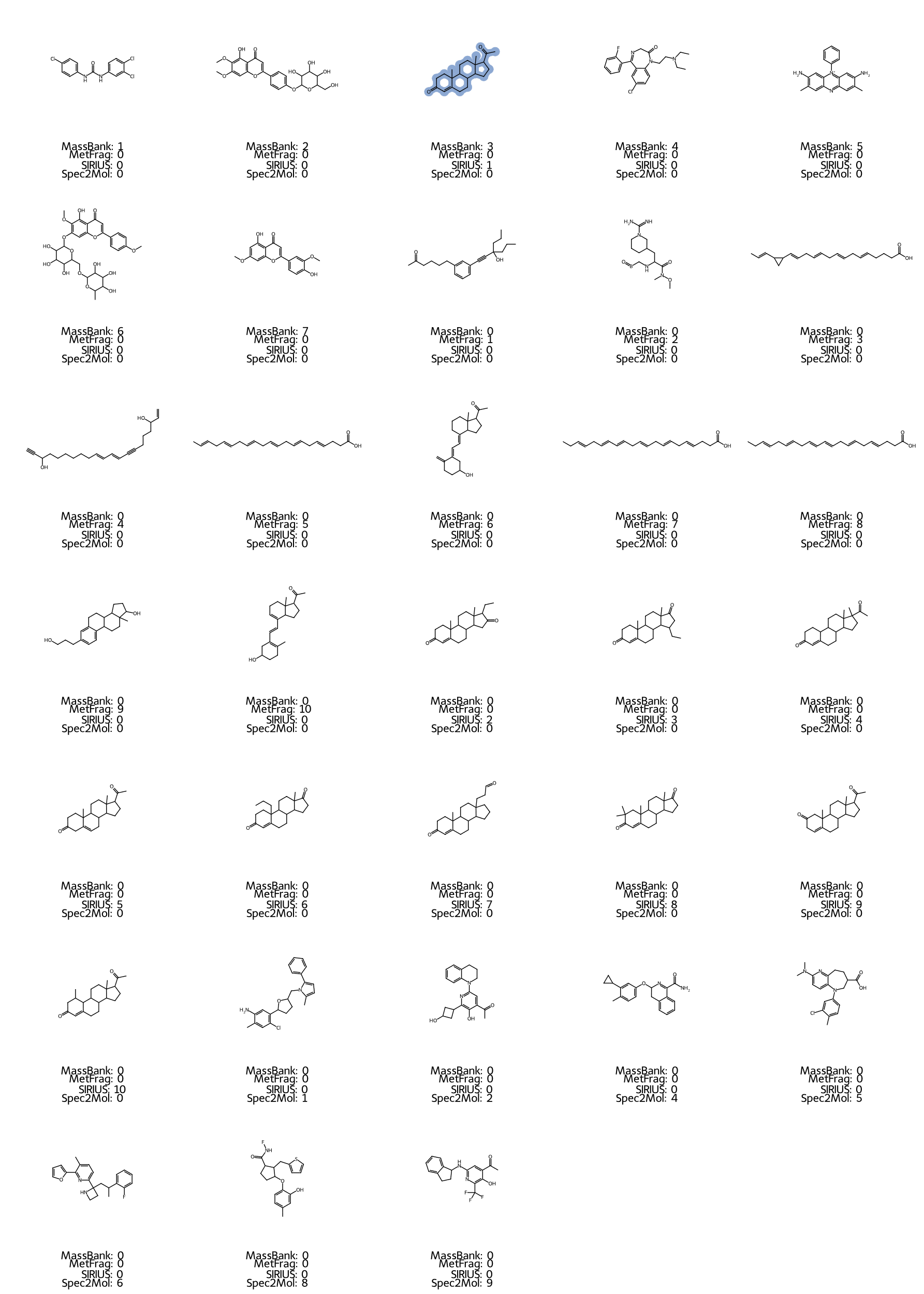


**Figure S12** Candidate structures for spiked feature with *m/z* 315.2322 and rank in annotation method. The correct structure is highlighted in blue.


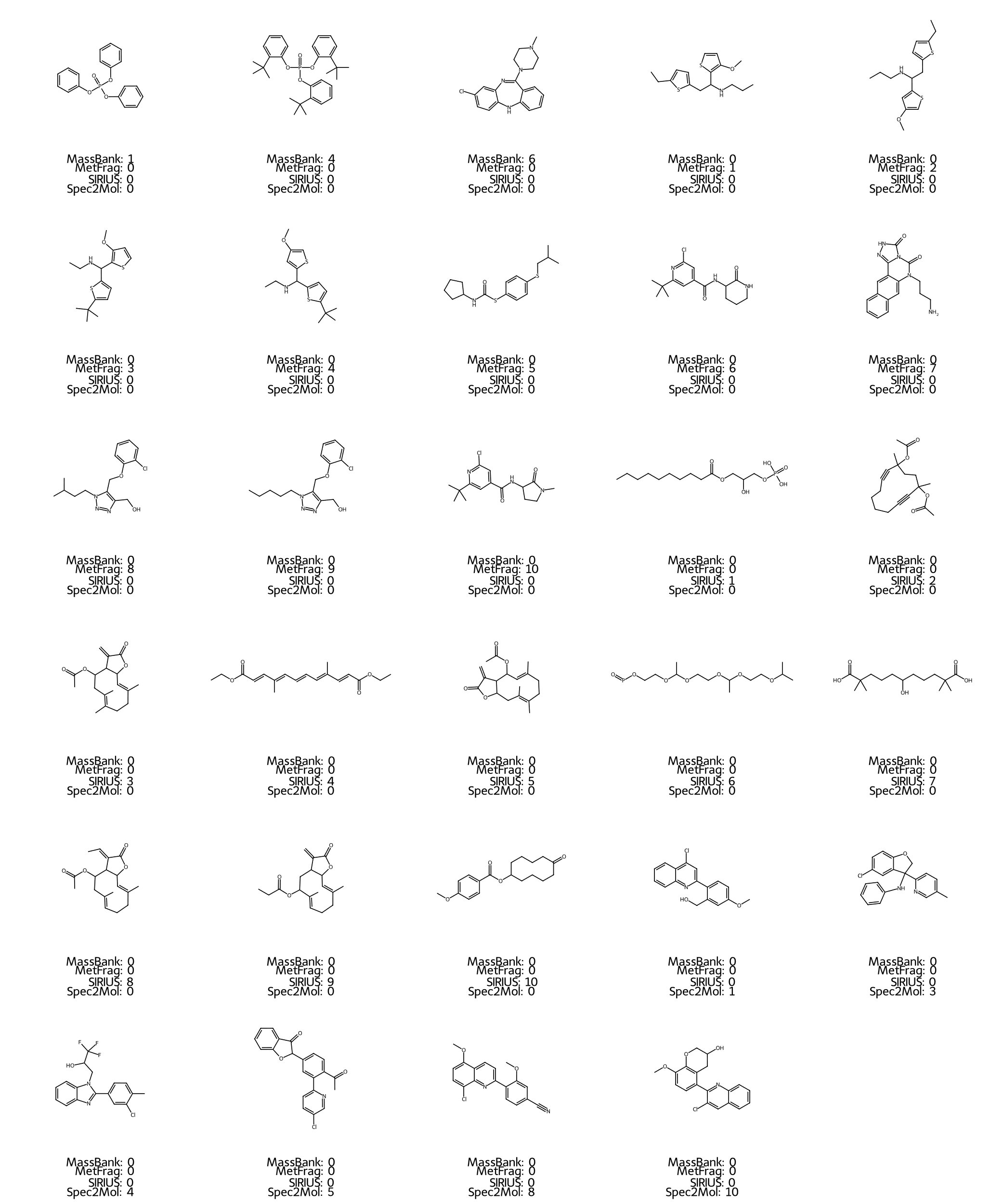


**Figure S13** Candidate structures for unknown feature with *m/z* 327.1565 and rank in annotation method.


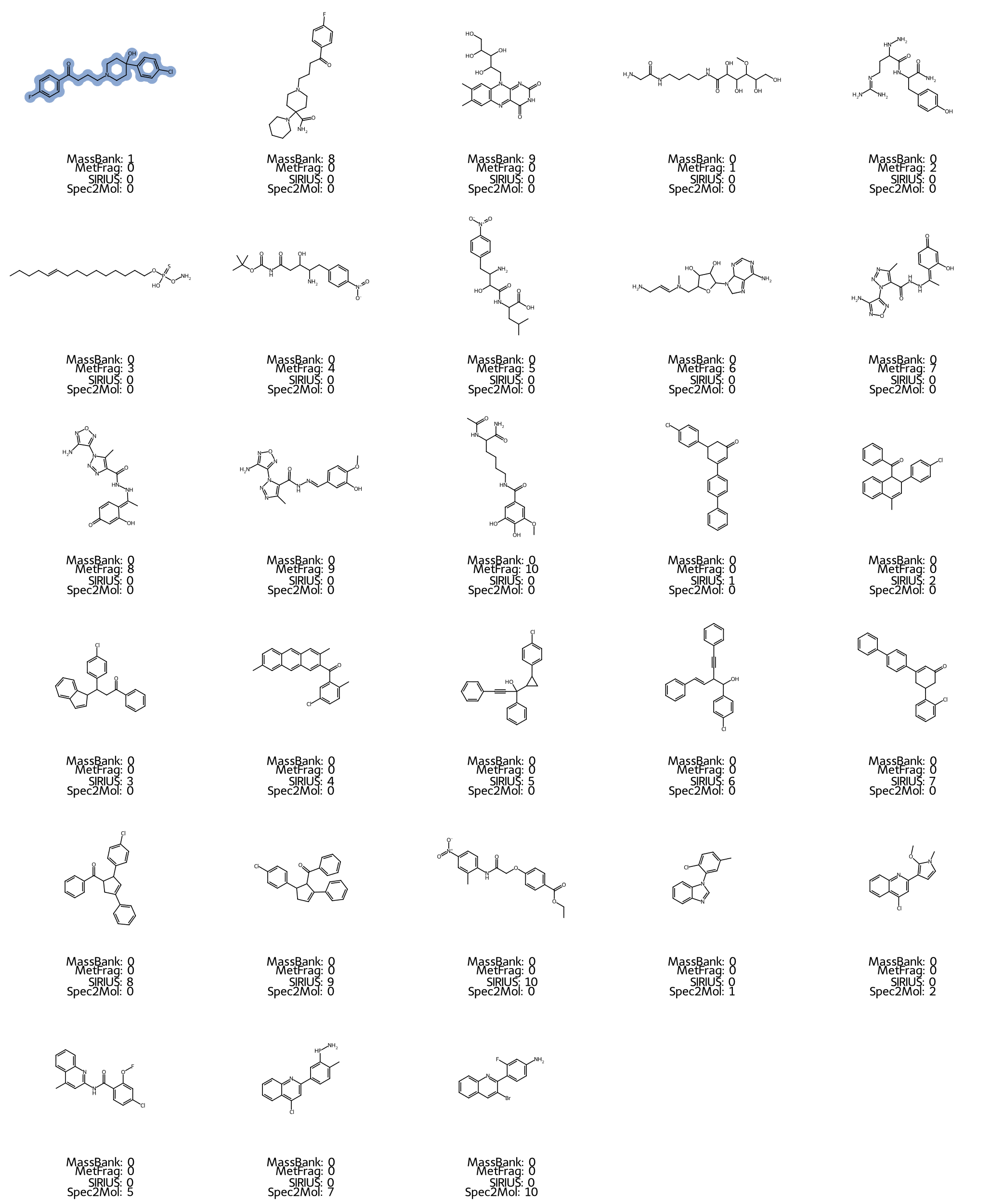


**Figure S14** Candidate structures for spiked feature with *m/z* 376.1487 and rank in annotation method. The correct structure is highlighted in blue.


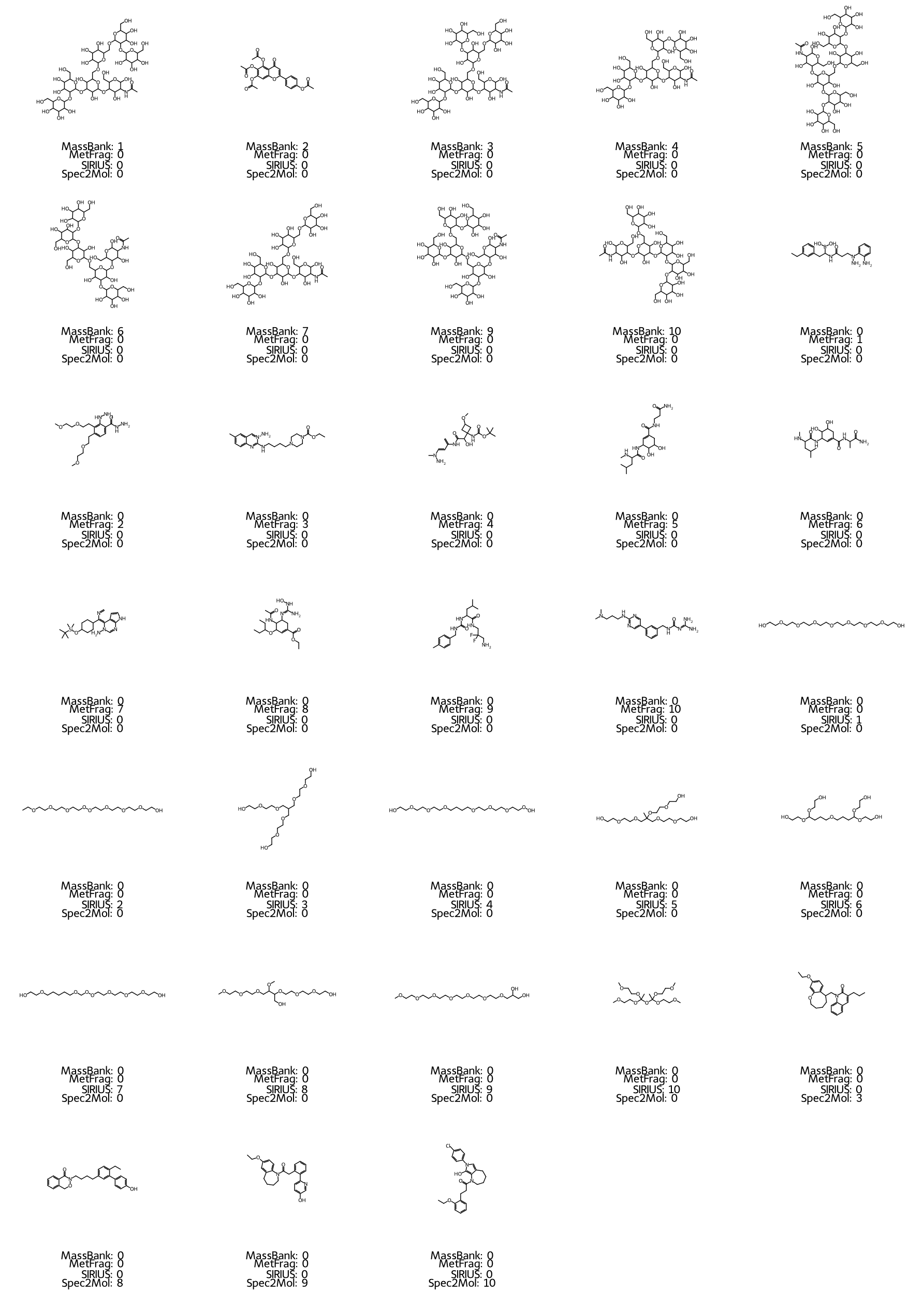


**Figure S15** Candidate structures for unknown feature with *m/z* 388.2547 and rank in annotation method.


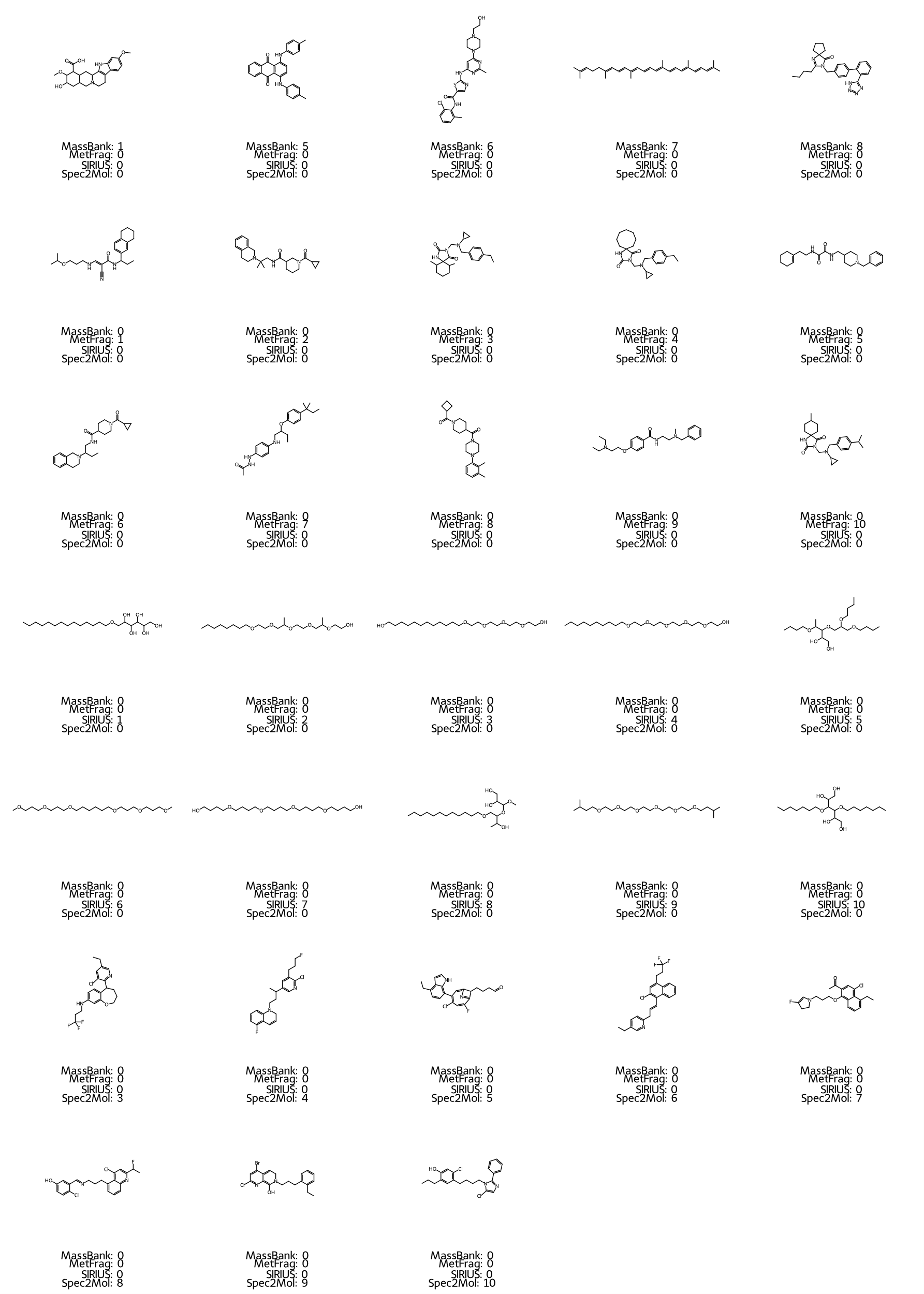


**Figure S16** Candidate structures for unknown feature with *m/z* 401.2872 and rank in annotation method.


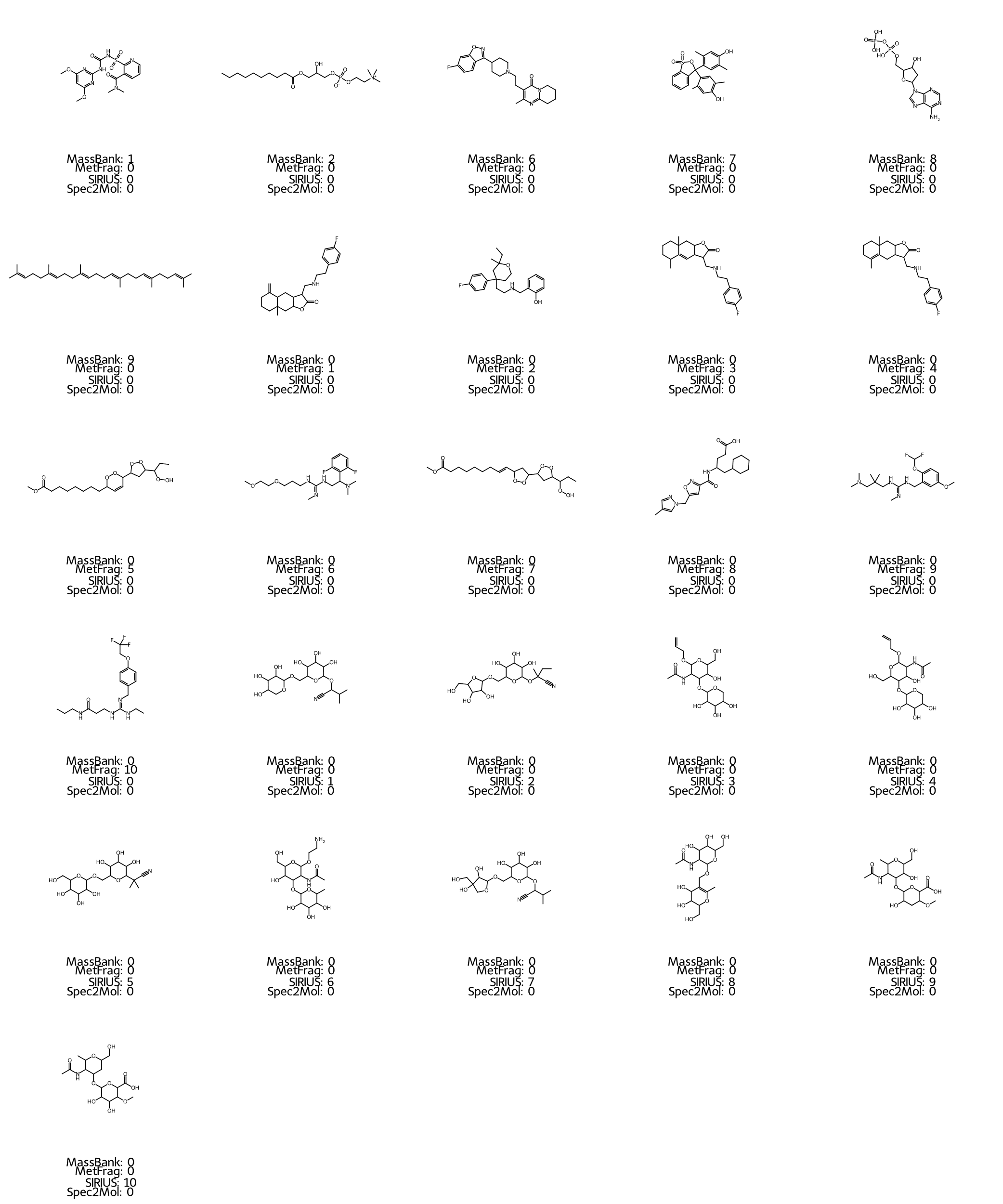


**Figure S17** Candidate structures for spiked feature with *m/z* 411.1984 and rank in annotation method.


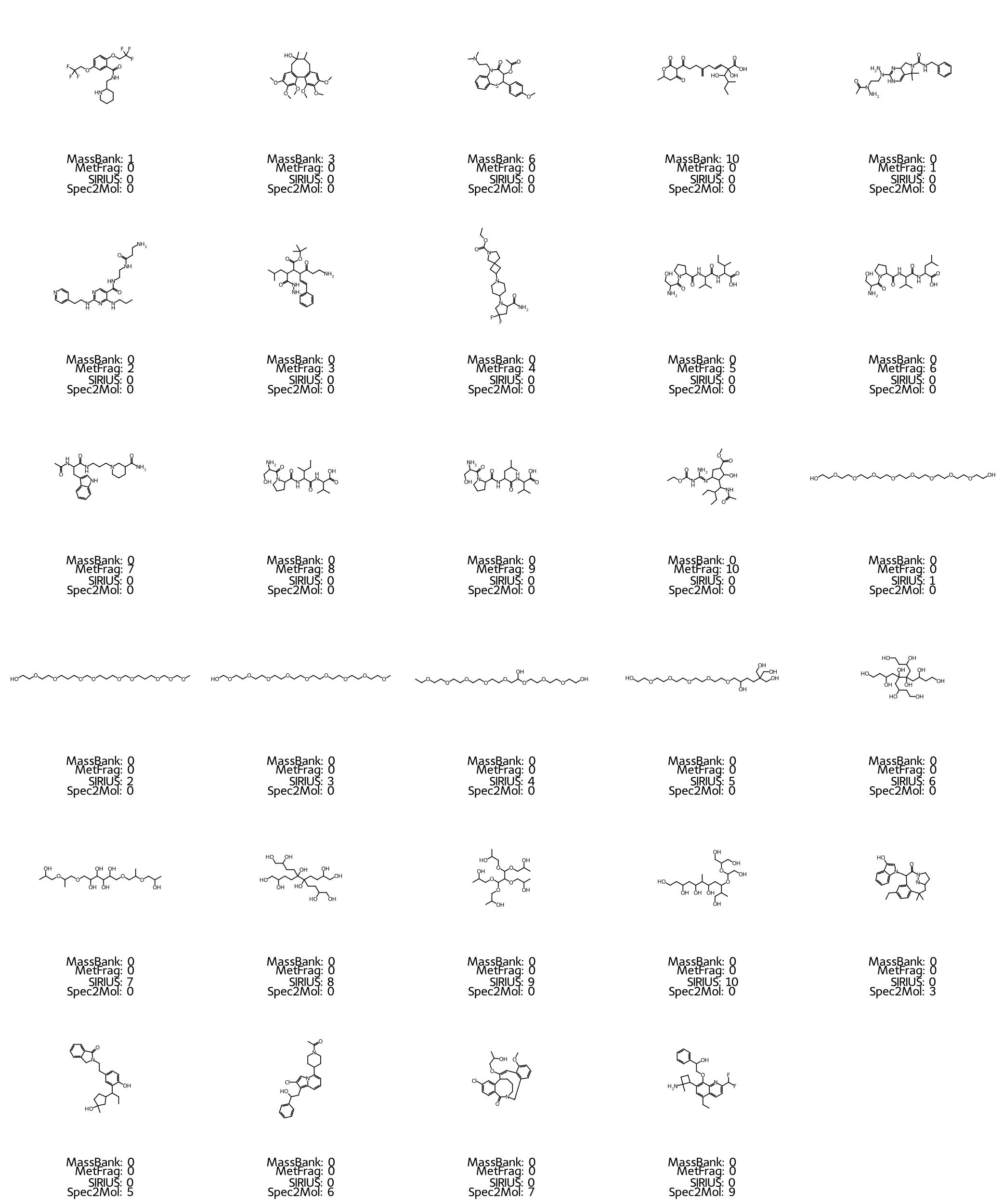


**Figure S18** Candidate structures for unknown feature with *m/z* 432.2812 and rank in annotation method.


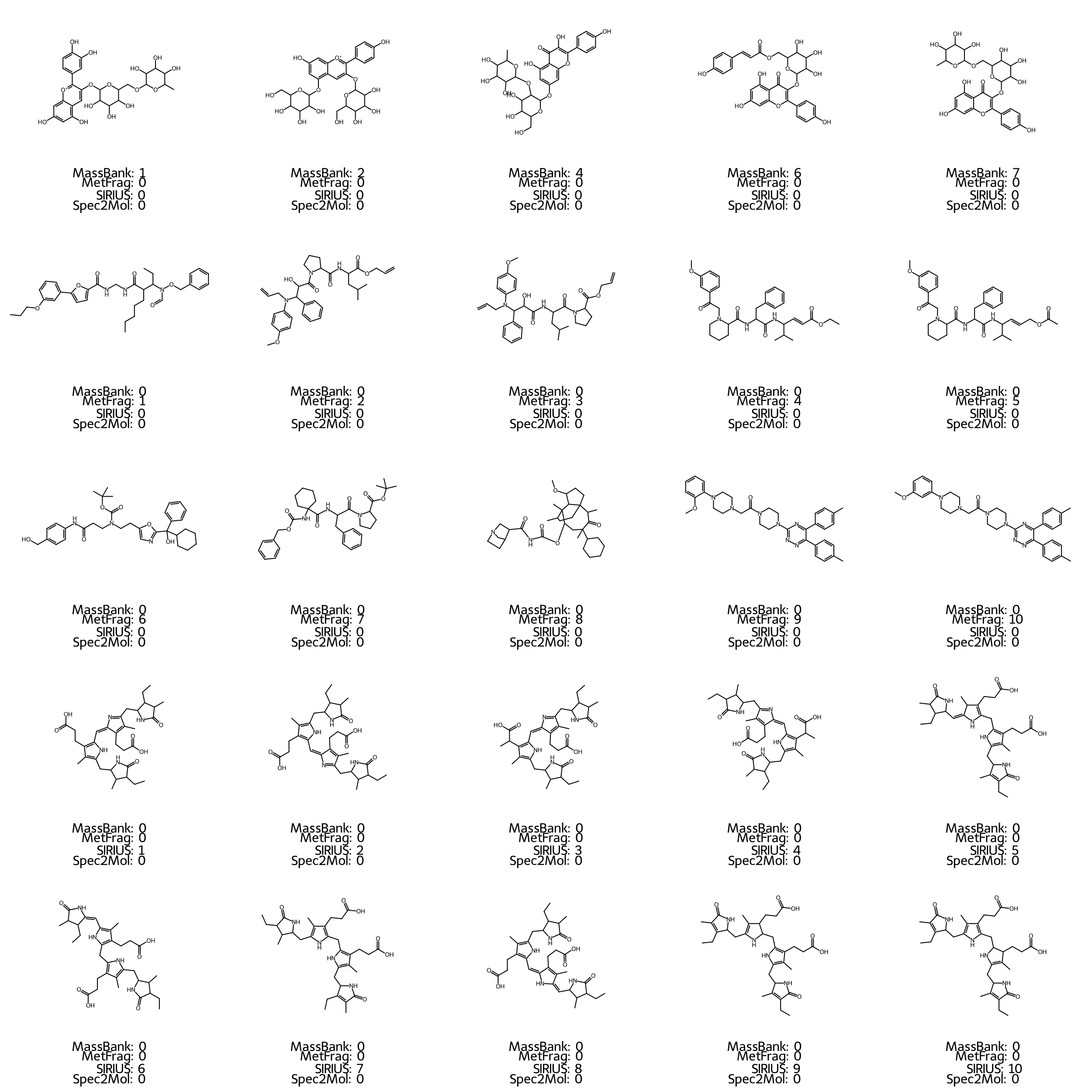


**Figure S19** Candidate structures for unknown feature with *m/z* 595.3495 and rank in annotation method.


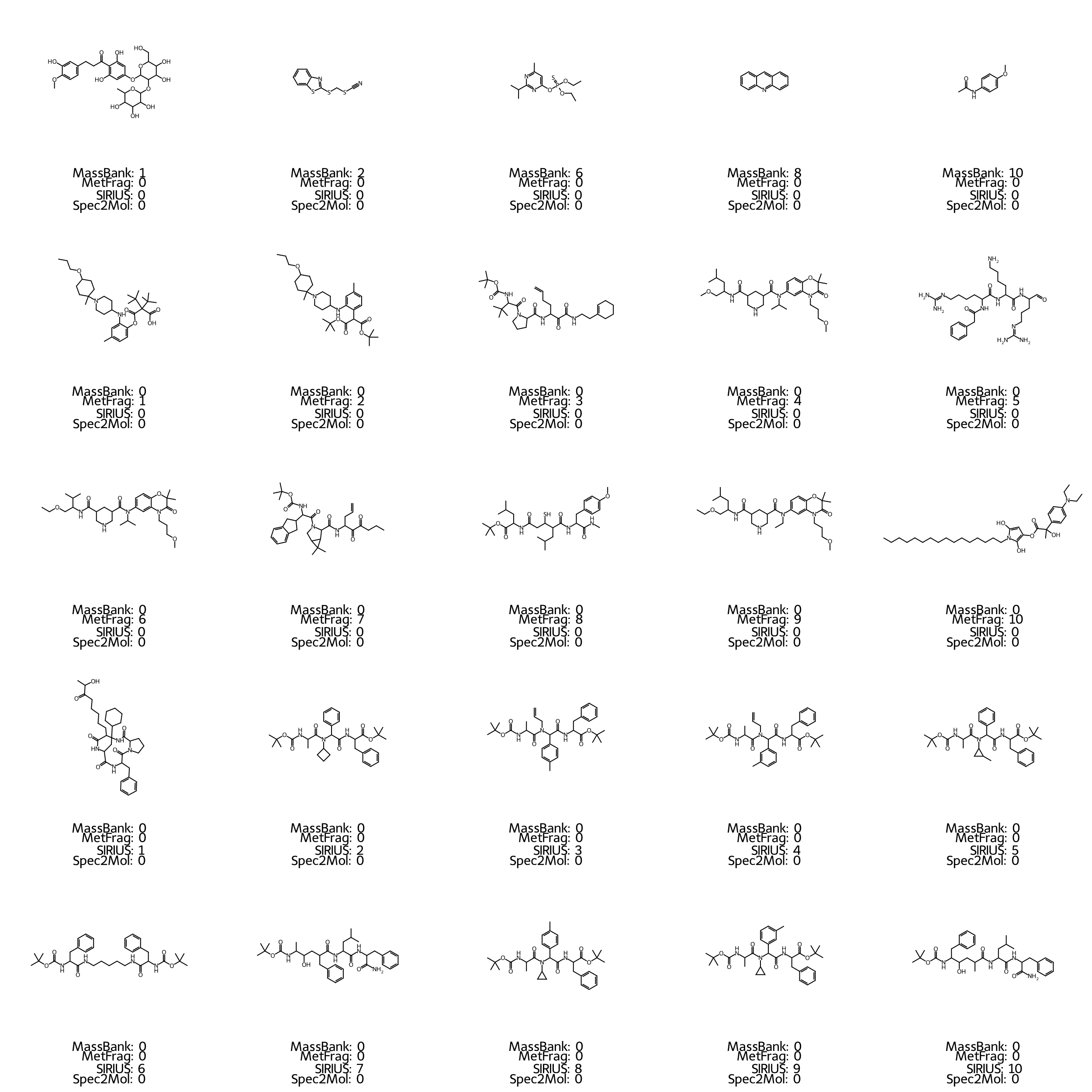


**Figure S20** Candidate structures for unknown feature with *m/z* 597.3648 and rank in annotation method.


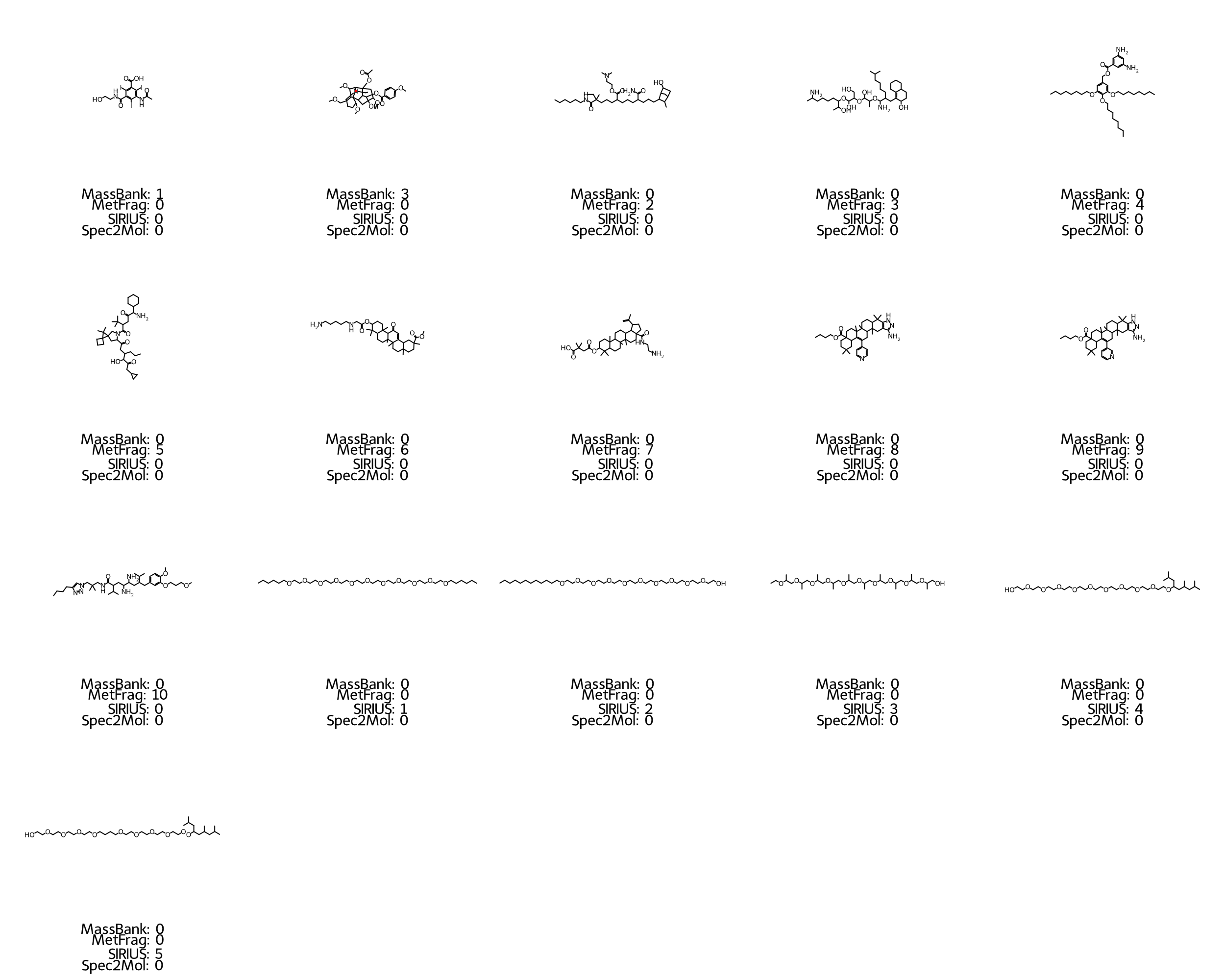


**Figure S21** Candidate structures for unknown feature with *m/z* 644.4951 and rank in annotation method.


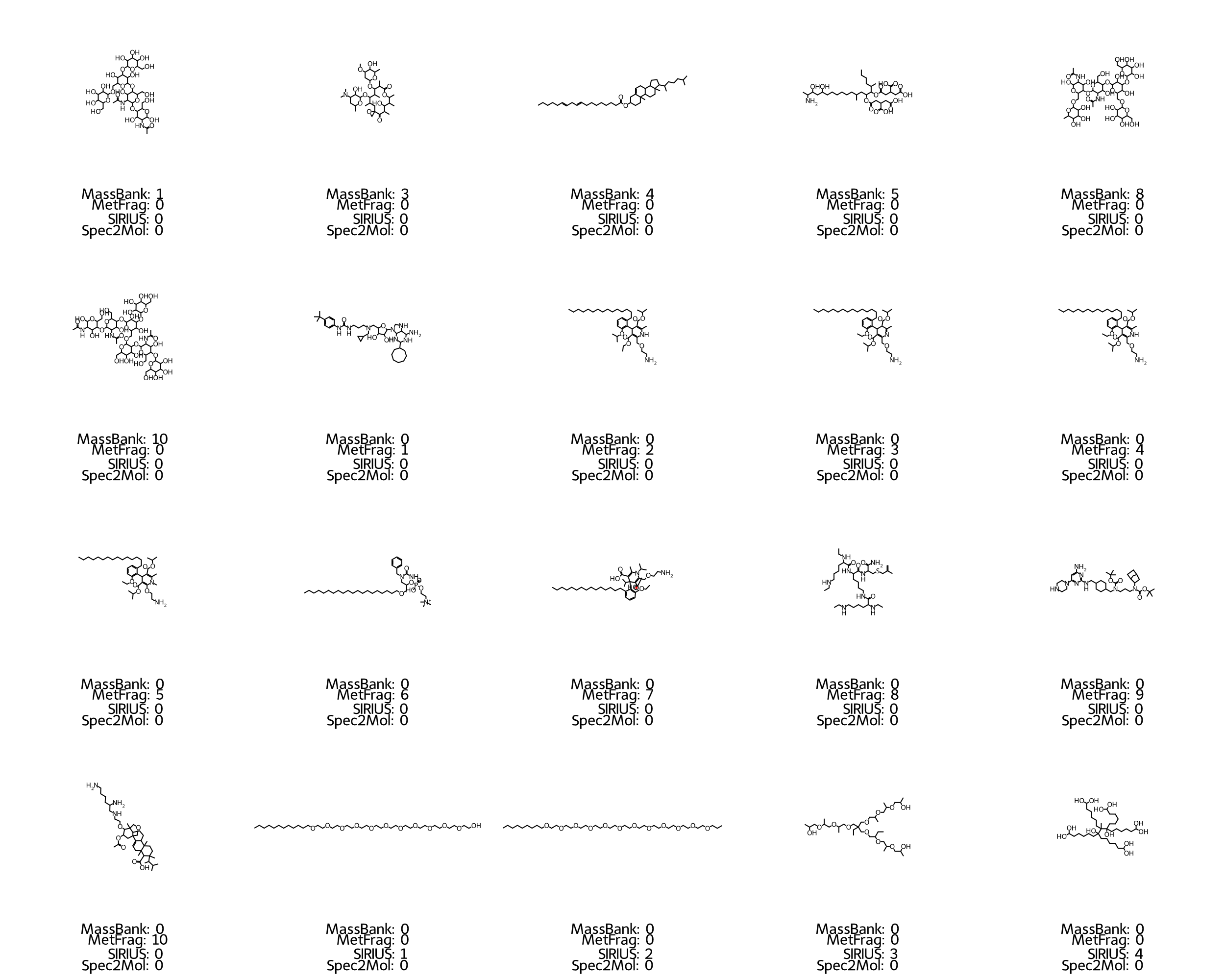


**Figure S22** Candidate structures for unknown feature with *m/z* 688.5216 and rank in annotation method.


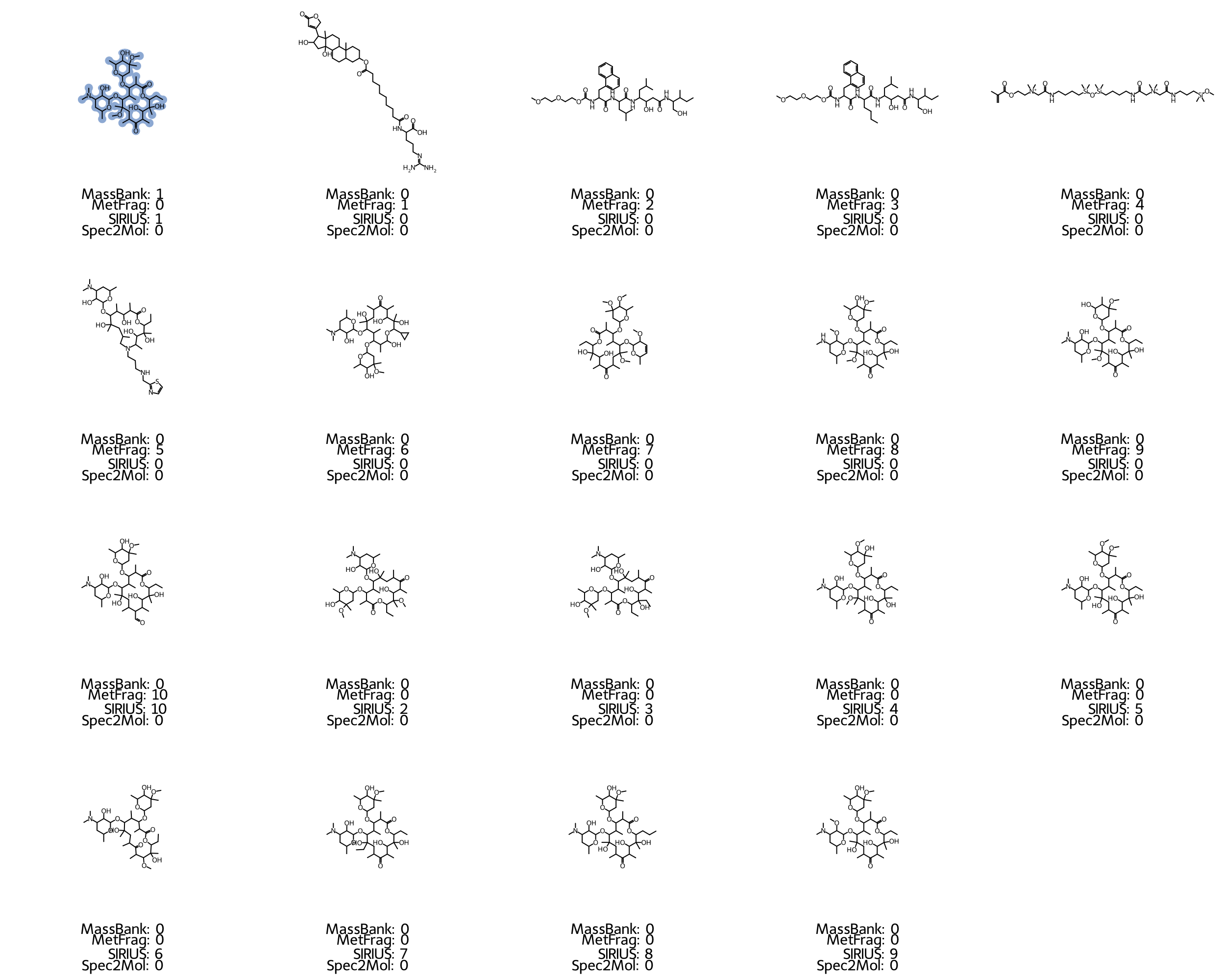


**Figure S23** Candidate structures for spiked feature with *m/z* 748.4870 and rank in annotation method. The correct structure is highlighted in blue.

## References

1. Dührkop K, Fleischauer M, Ludwig M, Aksenov AA, Melnik AV, Meusel M, Dorrestein PC, Rousu J, Böcker S (2019) SIRIUS 4: a rapid tool for turning tandem mass spectra into metabolite structure information. Nat Methods 16:299–302. https://doi.org/10.1038/s41592-019-0344-8

2. SIRIUS | Lehrstuhl Bioinformatik Jena. https://bio.informatik.uni-jena.de/software/sirius/. Accessed 30 Apr 2024

3. hclust function - RDocumentation. https://www.rdocumentation.org/packages/stats/versions/3.6.2/topics/hclust. Accessed 30 Apr 2024

4. Retention Time Indices Platform. http://rti.chem.uoa.gr/. Accessed 30 Apr 2024

5. Aalizadeh R, Alygizakis NA, Schymanski EL, Krauss M, Schulze T, Ibáñez M, McEachran AD, Chao A, Williams AJ, Gago-Ferrero P, Covaci A, Moschet C, Young TM, Hollender J, Slobodnik J, Thomaidis NS (2021) Development and Application of Liquid Chromatographic Retention Time Indices in HRMS-Based Suspect and Nontarget Screening. Anal Chem 93:11601–11611. https://doi.org/10.1021/acs.analchem.1c02348

6. sklearn.model_selection.train_test_split. In: Scikit-Learn. https://scikit-learn/stable/modules/generated/sklearn.model_selection.train_test_split.html. Accessed 30 Apr 2024

7. scikit-learn: machine learning in Python — scikit-learn 1.4.2 documentation. https://scikit-learn.org/stable/index.html. Accessed 30 Apr 2024

8. sklearn.preprocessing.MinMaxScaler. In: Scikit-Learn. https://scikit-learn/stable/modules/generated/sklearn.preprocessing.MinMaxScaler.html. Accessed 30 Apr 2024

9. MassBank consortium and its contributors (2023) MassBank/MassBank-data: Release version 2023.11

10. Ruttkies C, Schymanski EL, Wolf S, Hollender J, Neumann S (2016) MetFrag relaunched: incorporating strategies beyond in silico fragmentation. J Cheminformatics 8:3. https://doi.org/10.1186/s13321-016-0115-9

11. MetFrag - home. https://ipb-halle.github.io/MetFrag/. Accessed 30 Apr 2024

12. Your SIRIUS Documentation. In: SIRIUS Doc. https://boecker-lab.github.io/docs.sirius.github.io/. Accessed 30 Apr 2024

13. Lawson TN, Weber RJM, Jones MR, Chetwynd AJ, Rodrı́guez-Blanco G, Di Guida R, Viant MR, Dunn WB (2017) msPurity: Automated Evaluation of Precursor Ion Purity for Mass Spectrometry-Based Fragmentation in Metabolomics. Anal Chem 89:2432–2439. https://doi.org/10.1021/acs.analchem.6b04358

14. GitHub - computational-metabolomics/msPurity: R-package - Automated Evaluation of Precursor Ion Purity for Mass Spectrometry Based Fragmentation in Metabolomics. https://github.com/computational-metabolomics/msPurity?tab=readme-ov-file. Accessed 30 Apr 2024

15. Litsa EE, Chenthamarakshan V, Das P, Kavraki LE (2023) An end-to-end deep learning framework for translating mass spectra to de-novo molecules. Commun Chem 6:1–12. https://doi.org/10.1038/s42004-023-00932-3

16. GitHub - KavrakiLab/Spec2Mol. https://github.com/KavrakiLab/Spec2Mol. Accessed 30 Apr 2024

17. Aalizadeh R, Thomaidis NS, Bletsou AA, Gago-Ferrero P (2016) Quantitative Structure–Retention Relationship Models To Support Nontarget High-Resolution Mass Spectrometric Screening of Emerging Contaminants in Environmental Samples. J Chem Inf Model 56:1384–1398. https://doi.org/10.1021/acs.jcim.5b00752

18. Collision Cross Section Database and Prediction. https://ccsbase.net/. Accessed 30 Apr 2024

19. Ross DH, Cho JH, Xu L (2020) Breaking Down Structural Diversity for Comprehensive Prediction of Ion-Neutral Collision Cross Sections. Anal Chem 92:4548–4557. https://doi.org/10.1021/acs.analchem.9b05772

20. PubChemLite for Exposomics. https://zenodo.org/records/4183801. Accessed 30 Apr 2024

21. GitHub - dylanhross/c3sdb. https://github.com/dylanhross/c3sdb. Accessed 30 Apr 2024

22. RDKit. https://www.rdkit.org/. Accessed 30 Apr 2024

23. rdkit.Chem.rdchem module — The RDKit 2024.03.1 documentation. https://www.rdkit.org/docs/source/rdkit.Chem.rdchem.html. Accessed 30 Apr 2024

24. rdkit.Chem.SaltRemover module — The RDKit 2024.03.1 documentation. https://www.rdkit.org/docs/source/rdkit.Chem.SaltRemover.html. Accessed 30 Apr 2024

25. GitHub - baoilleach/nocharge: Attempt to neutralize all atoms with +1 or -1 charge in a molecule. https://github.com/baoilleach/nocharge. Accessed 30 Apr 2024

26. rdkit.Chem.MolStandardize.rdMolStandardize module — The RDKit 2024.03.1 documentation. https://www.rdkit.org/docs/source/rdkit.Chem.MolStandardize.rdMolStandardize.html. Accessed 30 Apr 2024

27. rdkit.Chem.rdmolops module — The RDKit 2024.03.1 documentation. https://www.rdkit.org/docs/source/rdkit.Chem.rdmolops.html. Accessed 30 Apr 2024

28. McInnes L, Healy J, Melville J (2020) UMAP: Uniform Manifold Approximation and Projection for Dimension Reduction

29. CRAN - Package rcdk. https://cran.r-project.org/web/packages/rcdk/index.html. Accessed 30 Apr 2024

30. UMAP: Uniform Manifold Approximation and Projection for Dimension Reduction — umap 0.5 documentation. https://umap-learn.readthedocs.io/en/latest/. Accessed 30 Apr 2024

31. rdkit.DataStructs.cDataStructs module — The RDKit 2024.03.1 documentation. https://www.rdkit.org/docs/source/rdkit.DataStructs.cDataStructs.html. Accessed 30 Apr 2024
